# A Comprehensive Review on Optimal Welding Conditions for Friction Stir Welding of Thermoplastic Polymers and Their Composites

**DOI:** 10.3390/polym13081208

**Published:** 2021-04-08

**Authors:** Syed Haris Iftikhar, Abdel-Hamid Ismail Mourad, Jamal Sheikh-Ahmad, Fahad Almaskari, S. Vincent

**Affiliations:** 1 Department of Mechanical Engineering, United Arab Emirates University, Al Ain 15551, United Arab Emirates; sharisiftikhar@gmail.com; 2 Department of Mechanical Engineering, Khalifa University of Science and Technology, Abu Dhabi 127788, United Arab Emirates; jamal.ahmad@ku.ac.ae; 3 Department of Aerospace Engineering, Khalifa University of Science and Technology, Abu Dhabi 127788, United Arab Emirates; fahad.almaskari@ku.ac.ae; 4 Department of Mechanical Engineering, Birla Institute of Technology and Science Pilani, Dubai Campus, Dubai 345055, United Arab Emirates; vincent@dubai.bits-pilani.ac.in

**Keywords:** friction stir welding, friction stir spot welding, polymers, polymer composites, review, thermoplastics

## Abstract

Friction stir welding (FSW) and friction stir spot welding (FSSW) techniques are becoming widely popular joining techniques because of their increasing potential applications in automotive, aerospace, and other structural industries. These techniques have not only successfully joined similar and dissimilar metal and polymer parts but have also successfully developed polymer-metallic hybrid joints. This study classifies the literature available on the FSW and FSSW of thermoplastic polymers and polymer composites on the basis of joining materials (similar or dissimilar), joint configurations, tooling conditions, medium conditions, and study types. It provides a state-of-the-art and detailed review of the experimental studies available on the FSW and FSSW between similar thermoplastics. The mechanical properties of FSW (butt- and lap-joint configurations) and FSSW weld joints depend on various factors. These factors include the welding process parameters (tool rotational speed, tool traverse speed, tool tilt angle, etc.), base material, tool geometry (pin and shoulder size, pin profile, etc.) and tool material, and medium conditions (submerged, non-submerged, heat-assisted tooling, cooling-assisted tooling). Because of the dependence on many factors, it is difficult to optimize the welding conditions to obtain a high-quality weld joint with superior mechanical properties. The general guidelines are established by reviewing the available literature. These guidelines, if followed, will help to achieve high-quality weld joints with least defects and superior mechanical properties. Apart from parametric-based studies, the statistical-based studies (e.g., analysis of variance (ANOVA)-based studies) are covered, which helps with the determination of the influential parameters that affect the FSW and FSSW weld joint strength. Also, the optimal ranges of the most influential process parameters for different thermoplastic materials are established. The current work on the development of general guidelines and determination of influential parameters and their operating ranges from published literature can help with designing smart future experimental studies for obtaining the global optimum welding conditions. The gaps in the available literature and recommendations for future studies are also discussed.

## 1. Introduction

Thermoplastics and their composites are becoming popular due to their high stiffness and strength-to-weight ratio. They have many industrial applications, like in automotive industries [1], to reduce the overall weight of the automobiles, in order to reduce their carbon footprint. These materials provide good insulating properties and are highly chemical resistant, and by the virtue of these qualities they are gaining popularity in piping and tank construction industries. There are many types of thermoplastic polymers available, and polyethylene is the most used one [2]. The widespread use of thermoplastics and their composites for industrial applications would require the fabrication of large thermoplastic-based components, which is not a feasible option. The extensive use of the thermoplastic structure demands its investigation for several types of joints: butt-, lap-, t-joint, etc. Several techniques are being explored for joining components made of thermoplastics and their composites, to create engineering structures [3]. These techniques include, but are not limited to, fusion welding, friction welding, vibration welding, adhesive joining, etc. However, among all these techniques, friction stir welding has a huge potential because of its low-cost equipment, low energy usage, better weld-strength joints, and in automation of the process for mass manufacturing [4].

**Figure 1:**
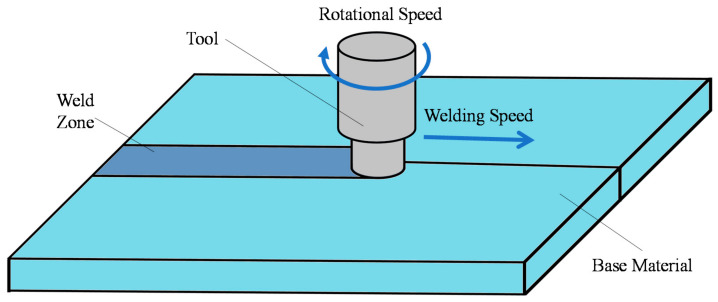
A schematic of the fundamental working process of the friction stir welding (FSW).

Friction stir welding (FSW), a comparatively new joining technique, was patented by The Welding Institute (TWI) in 1991 [5]. In this technique, the components are joined with the help of the frictional heat formed amongst a rapidly rotating tool and the joining workpieces, as shown in Figure 1. Although first developed for Al-based alloys [6], the technique has been successfully used to join other metals (Cu [7], Ti [8]) and alloys (Mg alloys [9], steel alloys [10]). Recently, its application has been extended to thermoplastic polymers and thermoplastic-based polymer composites [11]. The friction stir welding technique for polymers was patented in 2004 by Nelson et al. [12]. The friction stir spot welding (FSSW), a variant of the FSW technique, was developed in 2001 to replace the existing spot-welding technologies like resistance spot welding used for joining aluminum sheets in automotive industries [13]. This techniques has been successfully applied to different metallic materials (Al [14], Al alloys [15], Mg alloys [16,17], steels alloys [18,19]) and to thermoplastic materials (polypropylene [20], polycarbonate [21]).

Several review articles, overview articles, and book chapters have been written on the FSW of thermoplastic polymers and polymer composites, and their research highlights are summarized in Table 1. Several thermoplastic materials (PETG [22], ABS [23], PE [24,25,26], PA6 [27], PC [28], PP [29,30]) and their composites [31,32,33] have been studied. The feasibility of the FSW and FSSW techniques for thermoplastic materials have been assessed in comparison to other joining methods by several earlier studies. Strand [34] has made an early comparison of the capability of FSW of polymers with other established polymer joining techniques. The few simple processing steps, no part preparation, less processing time, no consumable materials, and low-cost machine and tools, capability of both continuous and discrete welding, and high repeatability of producing joints with high efficiencies make FSW a very able competitor of the already established techniques. Oliveira et al. [35] have studied the preliminary feasibility of FSSW for thermoplastics. Using PMMA (polymethyl-methacrylate) sheets, and they have shown that the FSSW technique is comparable (in strength and total joining time) to other processing techniques like microwave welding, thermal bonding, and ultrasonic welding.

Apart from FSW and FSSW, new variants of these techniques have also been developed for joining thermoplastic materials. The friction stir processing (FSP) technique is used for welding and processing of thermoplastics by reinforcing the weld zone (processing zone) with particulates to develop composite weld joints [46,47], which helps to improve mechanical properties [48,49]. The friction spot welding (FSpW) technique has also been successfully used to join thermoplastic sheets [50]. Unlike FSSW, the FSpW technique does not generate the keyhole at the weld joint, and improves the joint strength by reducing the notch stress concentration effects [51,52]. Friction spot joining (FSpJ), a new variant of the FSpW, has the potential to develop thermoplastic-metallic hybrid welds [52].

According to the best of the authors’ knowledge, there is no detailed review article available in the published literature on the determination and optimization of the welding conditions for maximizing joint strength of FSW and FSSW of similar thermoplastic materials. The present study covers the literature on the experimental investigations of FSW and FSSW of similar thermoplastic sheets and pipes. A detailed comparison of the process parameters, tooling conditions, and medium conditions from the available studies has been presented for optimizing the welding conditions. As a result, general guidelines are developed, most significant parameters are determined, and narrow optimal operating ranges of the significant process parameters are established, which will help with developing superior quality and strength thermoplastic weld joints using FSW and FSSW techniques.

## 2. Classification of the Available Literature

**Figure 2:**
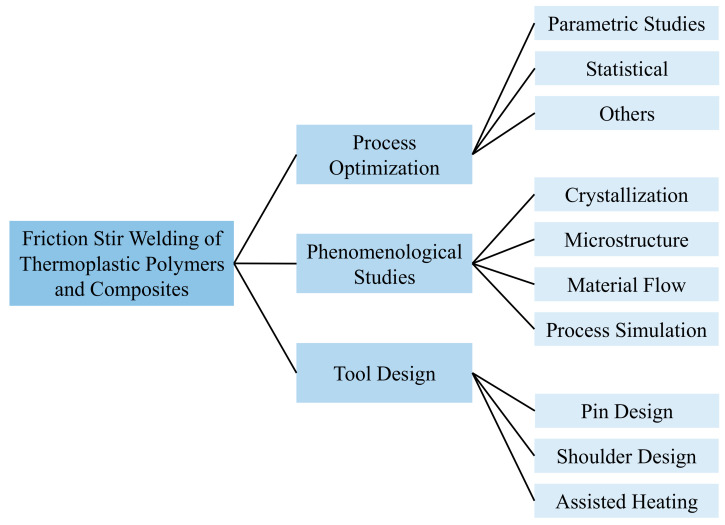
Classification of available literature.

The literature available on the FSW and FSSW of thermoplastic materials is vast and can be divided into several classes, as shown in Figure 2. The available studies can be categorized into the process optimization studies, phenomenological studies (mater flow, morphology, process simulation), and tool design studies. Most of the studies available are focused on welding of sheet materials, however, some studies have also been dedicated to pipes [53,54]. Both butt-joint and lap-joint configurations have been studied. Most of the studies are based on the welding of the thermoplastics processed using conventional techniques [55], however, some of them have also investigated three-dimensional (3D)-printed thermoplastics [56].

Many studies have analyzed the effects of process parameters, tooling (material and geometry), and medium conditions on the weld strength and quality. However, few of the studies have investigated the CFD (Computational Fluid Dynamics) modeling [57] and flow analysis [58] of the molten polymer flow behavior and weld morphology analysis [59], while several other studies have worked on the design and development of new tool technologies [60,61]. They include consumable tool technology for FSW [62,63] and FSSW [64]. Some of these studies have developed new measurement devices like the two-force extended octagonal ring dynamometer [65] and multi-axis force measurement dynamometer [66]. A few of these studies have designed the specialized platforms like robotic platforms [67] for effective FSW and FSSW of thermoplastic materials.

Many studies have investigated the welding of similar thermoplastic materials. However, there are several studies available which have investigated the FSW and FSSW techniques, and other variants of these techniques, for joining dissimilar materials. Some studies have investigated the welding between dissimilar thermoplastic materials. They include FSW of PE-PP [68], ABS-PC (non-submerged) [69], ABS-PC (submerged) [70], and HMWPE-PP [71,72]. Other similar studies include FSW of recycled HDPE-10Fe and LDPE-10Fe [73], FSW of HDPE through reinforcement of PP strips [74], and FSSW of PMMA-ABS [75]. Few of the studies have reinforced the weld joint between dissimilar thermoplastic materials with different particulates to improve mechanical properties [76]. They include FSW of PP-ABS sheets with copper nanopowder [77], and HDPE-ABS sheets with multiwalled carbon nanotubes [78,79]. Also, there are studies available on the development of thermoplastic-metallic hybrid welds. They include thermoplastic-metallic hybrid welds like PMMA-Cu [80], PP-Mg [81], PMMA-AA5058 Al alloy [82], PP-AA5052 Al alloy [83], and PET-A5052 Al alloy [84]. Other related studies include thermoplastic composite-metallic hybrid welds like CF-PPS/AA6181 Al alloy [85,86], CF-PPS/AA2024 Al alloy [87,88], and CF-PPS and GF-PPS/AZ31 Mg alloys [89]. There is a huge possibility of FSW on recycled composites [90,91].

This study will focus only on the review of the process optimization of the FSW and FSSW between similar thermoplastic polymers and polymer composites. The effect of different process parameters, tooling, and medium conditions on the weld joint mechanical properties will be covered in detail.

## 3. FSW Parametric Studies

Many studies are available on continuous and spot-welding forms of FSW. Both butt and lap configurations have been studied for continuous FSW. The FSSW is like resistance spot welding, which is a very widely used welding technique in the automotive industry and has many other promising applications. Most of the studies have optimized the welding conditions for maximizing the weld strength, as the structural components often fail at the weld joint.

### 3.1. Butt-Joint Configuration

The quality and strength of the butt-joint configuration weldments are assessed using joint tensile strength. Because of the large number of studies available on the butt-joint FSW, it is further categorized into three parts. First, we will investigate the studies which only consider the FSW process parameters. Then, we will study the combined effects of process parameters and tool geometry (pin profile and size). Finally, we will study the effects of external heat assistance along with process parameters and tooling conditions on the weld joint strength.

There are several studies available that have performed the butt-joint FSW of thermoplastic polymer and polymer composite sheets using a plain cylindrical pin profile tool. These studies help to only examine the parametric effects on the weld strength. Several thermoplastic sheets have been investigated, and their operating ranges and optimal values of the process parameters are summarized in Table 2. Apart from the single-side (single pass) butt-joint FSW studies, there are few studies available that have investigated the effects of double-side (double pass) butt-joint FSW on joint strength of thermoplastic polymers using the plain cylindrical pin profile tool. Arici and coworkers [92,93] have studied MDPE sheets. They have reported that the double-side FSW eliminates root defect, which has an important role in the initiation of joint failure [92]. Saeedy and Givi [94] have studied HDPE sheets. They have shown that the percent elongation, tensile strength, and impact strength are higher for double-side FSW compared to the single-side FSW. These studies suggest that it is beneficial to weld on both sides as it eliminates root defect and improves weld joint mechanical properties.

Many studies have also investigated the effects of pin profiles, along with processing parameters, on the joint mechanical properties of FSW of thermoplastic materials. The geometric profile of the tool pin is changed from plain cylindrical profile to further improve its overall mixing ability of the molten polymer material. Several thermoplastic sheets have been investigated using threaded cylindrical pin tools, which include HDPE (non-stationary [105,106] and stationary [107] shoulder tool), polyamide-6 [108], and polycarbonate [109]. Panneerselvam and Lenin [110] have studied the FSW of nylon-6 sheets using a left-handed threaded pin tool. They have shown that anticlockwise rotation provides better tensile strength, Shore-D hardness, Izod strength, and Charpy strength than the clockwise rotation of tool. They have suggested that a better joint with superior properties and less defects is achieved when the thread flute and pin rotation are not in the same directions, so, for a right-hand threaded pin with clockwise rotation or a left-hand threaded pin with anticlockwise rotation. Inaniwa et al. [111] have studied HDPE, polyamide-6, and polyvinyl chloride. They have shown that the joint strength of the materials increases with decreasing revolution pitch (revolution pitch = traverse speed/rotational speed), and that the suitable value of the revolution pitch increases with low melt viscosity materials. The suitable revolution pitch values are in the order PVC < HDPE < PA6, and the maximum joint strength efficiency was achieved for HDPE (70%), followed by PVC (45%) and PA6 (35%). Few of the studies have used other pin profile tools like frustum pin [112], tapered cylindrical pin [113], threaded conical pin [114], and milling tool with grooves [115].

Some of the studies have compared the joint strength obtained through cylindrical pin tools with other pin profile tools. Kaddour et al. [116] have studied HDPE sheets, and have shown that the cylindrical pin tool provides maximum tensile strength compared to the conical pin tool. Hoseinlaghab et al. [2] have also studied HDPE sheets, suggesting that the cylindrical pin achieved maximum creep-resistant welds (even more compared to the parent material) compared to the conical pin. Sadeghian and Givi [117] have studied ABS sheets, showing that the maximum tensile strength is achieved using the conical rather than the cylindrical pin tool. The two studies on HDPE suggest that the cylindrical pin is better than conical, while the study on ABS sheets suggests that the conical pin provides better results. Although, the reason behind the differing results could be the difference in properties of the base materials, like melt viscosity. However, the cylindrical pin has larger contact surface area with the molten polymer material than the conical pin, due to which it has improved mixing ability. This might be the reason behind the better performance of a cylindrical pin in comparison to a conical pin. Sahu et al. [118] have studied polypropylene sheets, and have shown that the square pin tool provides the maximum tensile strength compared to the cylindrical pin tool. This again might be because of its better mixing ability as the square edges are able to push more molten material than the plain cylindrical shape. Similarly, Pirizadeh et al. [119] have studied ABS sheets using a two-shoulder rotation-prevented tool (also called self-reacting tool; upper and bottom shoulders with a gap of about one-sheet thickness), with cylindrical and convex pin profiles. They have suggested that the new tool design eliminates root defects, and that the convex pin profile provides maximum tensile strength.

Few studies have investigated a larger set of varying pin profiles to study the effects of pin shape on joint strength in further detail. Payganeh et al. [120] have studied polypropylene-glass fiber (30 wt.%) sheets using triangular, threaded triangular, tapered with groove, and cylindrical with groove pin profile tools, suggesting that the tapered pin with groove profile provides the maximum tensile strength. Kordestani et al. [121] have studied polypropylene-carbon fiber (30 wt.%) and polypropylene-glass fiber (30 wt.%) sheets using square, threaded-tapered, threaded-tapered with a chamfer, and four-flute threaded pin profile tools, reporting that the maximum joint tensile and Izod impact strengths are provided by threaded-tapered pin with a chamfer. The above discussion narrows the best pin profiles for achieving maximum joint strength to square pin, convex pin, tapered pin with groove, and threaded-tapered pin with a chamfer. These four pin profiles need to be further studied together in detail to provide the best pin profile to achieve maximum weld joint strength.

Some of the studies have used special shoulder design tools, like double-step shoulder [122] and two-shoulder [123] tools, with threaded cylindrical pins. Romero et al. [124] have used a stationary shoulder tool (threaded cylindrical pin) and a conventional tool (threaded conical pin) for HDPE sheets. It has been reported that better quality welds with a lower number of visual defects are obtained using the stationary shoulder tool than with the conventional tool under similar operating conditions. Zafar et al. [125,126] have studied polyamide-6 sheets using a double shoulder tool with threaded pins. They have designed the double shoulder tool to control the molten nylon-6 flow as it has low viscosity. Through visual inspection and microscopic analysis of the weld zone, they have suggested that 300 rpm (low speed) rotational speed provides a defect-free weld joint.

The operating ranges and optimal values of the process parameters of all the above studies are summarized in Table 3.

Several studies have investigated the external heat-assisted tooling effects on the strength of the weld joint. Bagheri et al. [128] have studied ABS sheets using the fixed heated shoe tool with threaded pin. Moochani et al. [129] have studied polypropylene sheets using a heat-assisted stationary shoulder tool. Aydin [130] has studied pre-heated UHMWPE sheets using the threaded pin tool, and has shown that the joint tensile strength is improved with pre-heated sheets (89% efficiency) compared to non-preheated sheets (72% efficiency). Banjare et al. [131] have studied for polypropylene sheets using a heat-assisted tool with threaded cylindrical pin, and have suggested that the heat-assisted tooling provides better joint mechanical properties (tensile strength, elongation) compared to non-heated tooling conditions. Laieghi et al. [132] have studied Polyamide 6/Nitrile butadiene rubber (20 wt.%)–halloysite nantotubes nanocomposite sheets using a heat-assisted tool with stationary PTFE-coated shoulder and threaded pin. They have shown that the heat-assisted tool provides better weld quality and reduces defects and improves mechanical strength.

Some of the studies have suggested that the PTFE-coated tool shoulder further improves the weld joint mechanical properties [133,134,135]. Azarsa and coworkers [11,136] have studied HDPE sheets using a threaded pin tool with heat-assisted and PTFE-coated stationary shoulder. The weld defects and residual stress concentration are reduced and the surface quality and mechanical properties of the weld joint are improved by using the PTFE-coated tool shoulder because of the reduction in sticking of the polymer melt [136,137]. Mostafapour and Asad [138] have studied nylon-6 sheets using a heat-assisted tool with stationary PTFE-coated shoe, and have also suggested that PTFE coating improves the surface quality of the weld joint. Christy et al. [133] have also concluded that use of PTFE coatings improved the surface quality.

Some of the studies have considered other special tooling conditions. Mendes et al. [139] have studied robotic FSW for ABS sheets using a heat-assisted tool with stationary shoulder and conical threaded pin. Vijendra and Sharma [140] have studied HDPE sheets using an induction heat-assisted tool with taper-threaded pin, and have suggested that the heat-assisted welding melts and stirs the thermoplastic material easily. Nath et al. [141] have studied polypropylene sheets using a self-heated tool with threaded cylindrical pin, and have reported that the self-heated tool provides higher tensile strength and percent elongation of weld joints than with conventional tooling.

One of the studies investigated the weld nugget cooling effects on the joint mechanical properties. Nateghi and Hosseinzadeh [142] have investigated the effects of external cooling of the weld nugget using carbon-dioxide gas for HDPE sheets with a threaded pin tool. The joint mechanical properties improve (tensile strength increases, angular distortion decreases) with increasing the cooling pressure, which happens due to a drop in the thermal residual stresses. The operating ranges and optimal values of the process parameters of all the above studies are summarized in Table 4.

The above-detailed discussions suggest that the weld joint strength and quality of butt-joint FSW of thermoplastics depends on process parameters, tooling conditions (geometry, material, heating/cooling assistance), and medium conditions. Because of its dependence on many factors, only a few of them are considered in a single study. The available studies provide only the local optimum welding conditions, which is limited to the factors and their ranges considered. To determine the global optimum welding conditions, a smart and detailed experimentation plan needs to be devised [51]. The plan can be developed by using only the most influential parameters, which are discussed later in this study. This section will help with narrowing the operation range of these parameters. The use of only most influential parameters within their narrow operation ranges and the statistical techniques, like the Taguchi method, will help with the development of a detailed but smart experimentation plan to achieve the objectives with a lower number of experimentations. Ramanathan et al. [143] have also used the Taguchi optimization technique to narrow down the main parameters and to reduce the set of experiments. Also, based on the above discussions, some general guidelines can be developed which will help with achieving high-quality butt-joints with superior mechanical properties and less defects through FSW of thermoplastic materials. These guidelines will help in developing the experimentation plans for future studies in the field. The guidelines are:

- The double-side FSW technique should be used as it reduces defects and improves mechanical properties.
- The tool shoulder should be coated with PTFE as it reduces defects and improves surface quality and mechanical properties.
- The thermoplastic sheets should be pre-heated as it improves joint tensile strength.
- The heat-assisted tooling should be used as it improves joint mechanical properties.
- The cooling-assisted (weld nugget cooling) FSW technique should be used as it improves mechanical properties and reduces thermal residual stresses.
- The square pin, convex pin, tapered pin with groove, or threaded-tapered pin with a chamfer should be used instead of the plain cylindrical pin because of their better mixing ability of molten polymer material and for achieving better weld joint strengths.
- The stationary shoulder tool should be used as it provides better quality welds.
- For threaded pin profile, the tool should be designed such that thread flute and pin rotation are not in the same directions, so for a right-hand threaded pin with clockwise rotation or a left-hand threaded pin with anticlockwise rotation, to obtain better weld joint with better properties and less defects.

**Figure 3:**
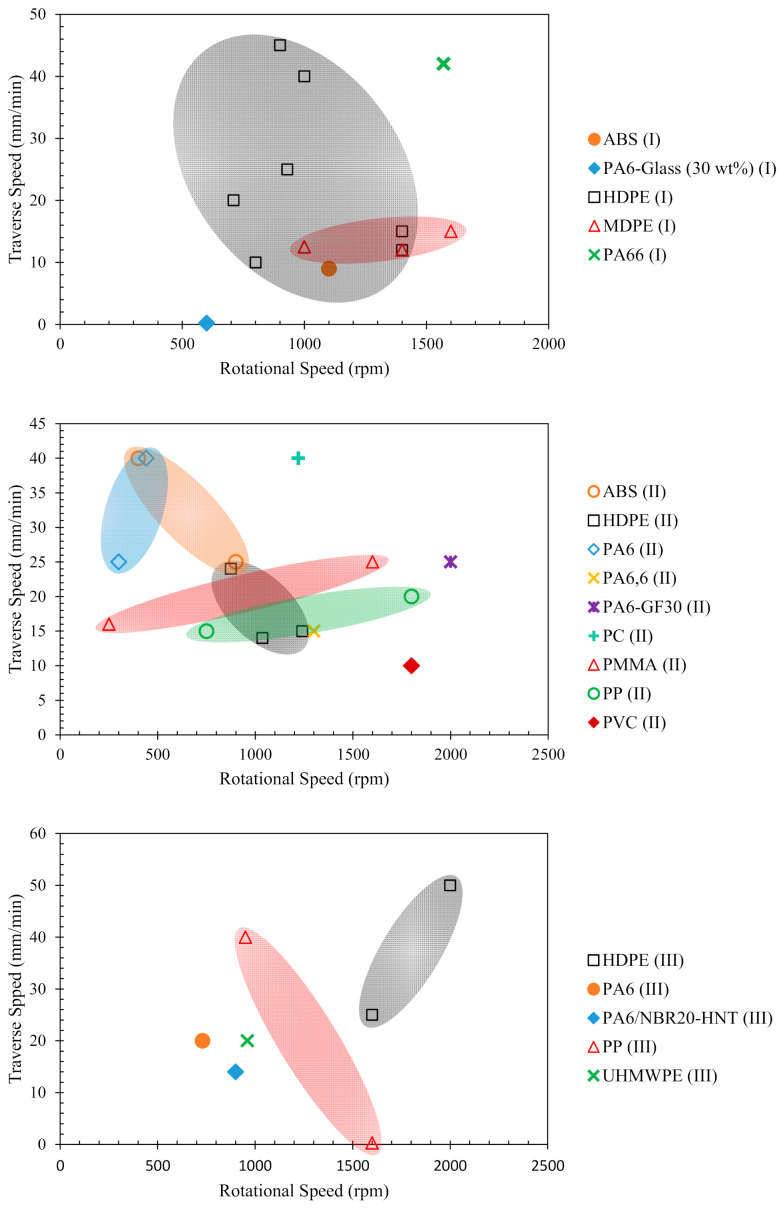
Optimal rotational and traverse speeds for achieving maximum joint tensile strength for FSW of thermoplastic sheets in butt-joint configuration. I denotes FSW (single- and double-side) using cylindrical pin profile, II denotes FSW with other than cylindrical pin profile, and III denotes heat-assisted FSW.

The FSW depends on many process parameters. However, as will be discussed in the later section, the rotational and traverse speeds are the most important parameters that affect the joint tensile strength. The range and average optimal rotational and traverse speeds for achieving maximum joint tensile strength from the Table 2, Table 3 and Table 4 are summarized in Table 5 and plotted in Figure 3.

There are very few studies available on FSW of pipes, because unlike other types of welding, there is complexity involved to move the high-speed rotating FSW tool along the curved path. Muñoz [144] has studied PE pipes which are used for natural gas applications. Vakili-Tahami et al. [123] have studied the creep behavior and have optimized the creep lifetime of friction stir-welded PMMA pipes.

Mosavvar et al. [127] have studied butt-joint FSW of HDPE pipes using a threaded cylindrical pin. The rotational speed was varied in a 1500–2500 rpm range, traverse speed in 110–150 mm/min range, and tool offset in 2.5–4.5 mm range. The maximum yield strength is obtained at the optimum parameter values of 2500 rpm, 110 mm/min, and 3.5 mm. This study suggests that it is possible to transfer knowledge gained from the vast studies available on sheets to the pipes, which has more potential applications. The rotational and traverse speeds are important parameters for FSW of thermoplastic sheets, so they are also investigated for pipes. Also, tilt angle is an important parameter for sheets, and it is used as tool offset for pipes. Composite pipes have revolutionized the industry at many fronts [145,146].

### 3.2. Lap-Joint Configuration

There are few studies available on FSW of thermoplastic polymer and polymer composite sheets in lap-joint configuration, and they are summarized in Table 6. The joint strength and quality for the lap-joint configuration is assessed using the tensile shear strength. Few of these studies have been carried out under non-submerged conditions. Ahmadi et al. [147] have studied polypropylene-carbon fiber (20%) composite sheets using varying tool pin profiles and have suggested that threaded cylindrical-conical pin profile provides the maximum joint strength. In another study, Ahmadi et al. [148] have investigated the same material using a grooved cylindrical-conical pin. Derazkola and Simchi [149] have studied PMMA sheets using several pin profiles, like cone, square, and triangle frustums. They have shown that the cone frustum provides the maximum joint strength. The above discussion narrows the best pin profiles for achieving maximum joint strength to threaded cylindrical-conical pin and cone frustum pin. These two pin profiles need to be further studied together in detail to provide the best pin profile to achieve the highest lap-joint strength. Some of the studies have been carried out under submerged conditions. Gao et al. [150] have studied HDPE sheets using threaded pin, and have reported that the maximum joint strength is 12.3 MPa for the submerged case at optimal operating conditions, while the joint strength under non-submerged case is 9.6 MPa under similar conditions. Yan et al. [151] have also studied HDPE sheets but using a double-pin tool. Few studies have also investigated the effects of polymeric-based tool shoulders. Eslami et al. [152] have studied polypropylene-polyethylene weld using a Teflon stationary shoulder tool. Also, Eslami et al. [153] have investigated various tool shoulder designs, and have shown for the polystyrene-polypropylene weld that the polymeric-based stationary tool shoulder provides the best weld quality.

Based on the above discussions, general guidelines can be developed which will help with achieving high-quality lap-joints with superior mechanical properties and less defects through friction stir welding of thermoplastic polymers and polymer composites. The guidelines are:

- The submerged (underwater) FSW technique should be used as it provides better joint strength than conventional FSW.
- The threaded cylindrical-conical pin and cone frustum pin profiles should be used as they provide better weld joint strength.

**Figure 4:**
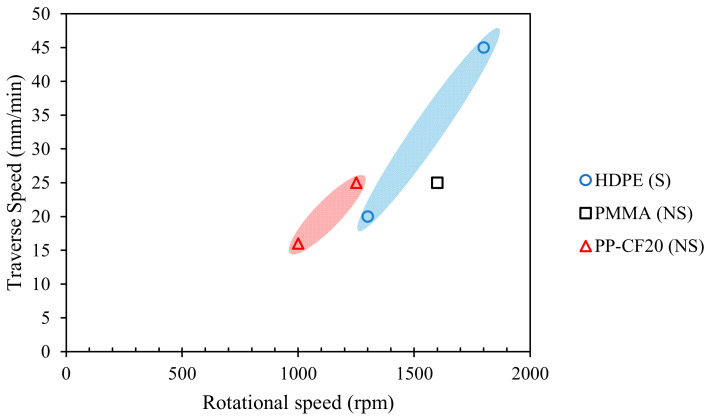
Optimal rotational and traverse speeds for achieving maximum joint strength for lap-joint FSW of thermoplastic sheets. NS = Non-Submerged, S = Submerged.

The lap-joint FSW depends on many process parameters. However, as will be discussed in a later section, the most significant parameters that affect the joint strength are traverse and rotational speeds. The optimal traverse and rotational speeds for achieving maximum joint strength from Table 6 are plotted in Figure 4. For HDPE, the optimal traverse speed is reported in the range of 20–45 mm/min, and rotational speed in the range of 1300–1800 rpm: the average of the reported traverse speed is 32.5 mm/min, and of the rotational speed is 1550 rpm. For PP-CF20, the optimal traverse speed is in the range of 16–25 mm/min (20.5 mm/min average traverse speed), with optimal rotational speed in the range of 1000–1250 rpm (1125 rpm average rotational speed). For PMMA, the reported optimal traverse speed is 25 mm/min and rotational speed is 1600 rpm.

The widespread use of the modern thermoplastic structures requires detailed investigation not for only butt- and lap-joints, but also for other types of joints, like T-joint. Most studies are available on butt-joints while few are on lap-joints, however, the studies on other types of joints are lacking. This research gap needs to be filled as other types of joints are also useful in automotive and other structural industries. Elyasi and Derazkola [154] have studied the T-joint FSW of PMMA experimentally and through the thermomechanical FE method. They have used a frustum pin profile tool and the tool was rotated in anti-clockwise direction. The rotational speed was varied from 1000 to 1600 rpm and traverse speed from 25 to 50 mm/min, with a constant tilt angle of 2° and plunge depth of 0.2 mm. They have shown that the maximum tensile and flexural strengths are obtained at 1600 rpm and 25 mm/min.

## 4. FSSW Parametric Studies

Several studies are available on FSSW of thermoplastic polymers and polymer composites. These studies investigate the parametric and tool geometry effects on the joint strength. The joint strength for this category of weldments is assessed using lap-shear tensile force, which is obtained through lap-shear tests. Some studies have not varied the tool pin profiles to only investigate the parametric effects on the weld strength. Lambiase et al. [155,156] have studied polycarbonate sheets using cylindrical pins. The tapered cylindrical tool pin profile has been used to weld HDPE [13,157], PP [158,159], and HDPE-glass hollow spheres composite [160] sheets. They have suggested that thin weld nuggets are produced using short dwell times which have low joint strengths, while thick weld nuggets are produced using longer dwell times which have high joint strengths [13]. They have also suggested that the weld joint strength is negligibly affected by the plunge rate.

Several studies have varied pin profiles and materials to investigate their effects and to determine the best pin profile and material which maximizes the joint strength. Yan et al. [161] have studied ABS sheets using a triflute pin, and have shown that the triflute pin provides better joint strength than a cylindrical pin. Lambiase et al. [162] have studied PC sheets using cylindrical and tapered pin profiles, and have shown that the cylindrical pin provides better joint strength. Bilici [163] have studied PP sheets using cylindrical (straight, tapered, threaded) and square pin profiles. They have shown that the tapered cylindrical pin provides maximum joint strength. Bilici and Yükler [164] have studied HDPE sheets using cylindrical (straight, tapered, threaded), hexagonal, square, and triangular pin profiles. They have reported that the pin profile significantly affects the joint strength, and that the maximum joint strength is obtained through the tapered cylindrical pin. Also, they have suggested that a tapered threaded pin will provide the best results. Bilici et al. [165] have studied HDPE sheets using tools made of several materials (316 stainless-steel, aluminum 1050, pure copper, and SAE 1020 steel) to examine the effects of heat transfer coefficient of tool material on weld strength. They have reported that the pure copper tool provides the best results. The operating ranges of the process parameters and the optimized process parameters of all the studies are summarized in Table 7.

Based on the above discussions, general guidelines can be developed which will help with achieving high-quality joints with superior mechanical properties and less defects through FSSW of thermoplastic materials. The guidelines are:

- The welding should be performed with longer dwell times as it improves joint strength.
- The effect of plunge rate should not be investigated in future studies, as it is not a significant parameter and has negligible effect on the weld joint strength.
- Tapered cylindrical and tapered threaded pin profiles should be used as they provide better weld joint strength.
- The tool should be made of pure copper material as it provides better results than aluminum and steel alloys.

**Figure 5:**
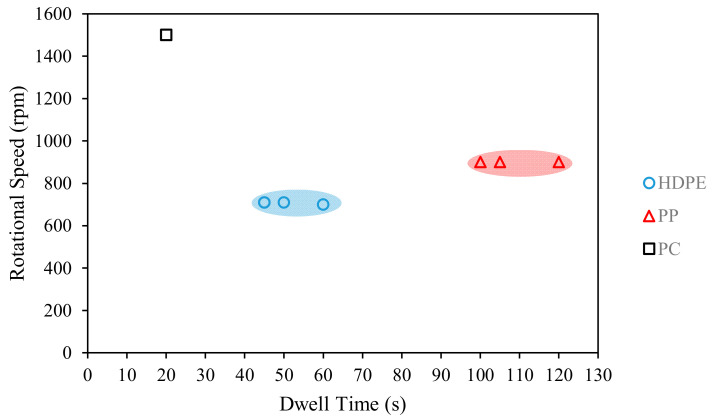
Optimal dwell times and rotational speeds for maximum joint strength of FSSW of thermoplastic sheets.

The FSSW of thermoplastic materials depends on many process parameters. However, as will be discussed in the next section, the dwell time and rotational speed are the most important parameters that affect the joint strength. The optimal dwell time and rotational speeds for achieving maximum joint strength from the Table 7 are plotted in Figure 5. For HDPE, the optimal dwell time is reported in the range of 45–60 s, and rotational speed in the range of 700–710 rpm: the average of the reported dwell time is 50 s, and of the rotational speed is 707.5 rpm. For PP, the optimal dwell time is in the range of 100–120 s (108.3 s average dwell time), with optimal rotational speed of 900 rpm. For PC, the optimal dwell time is 20 s and rotational speed is 1500 rpm.

## 5. Statistical-Based Studies

Several studies have conducted statistical analysis, like analysis of variance (ANOVA), for determining the most significant parameters that affect the weld joint mechanical properties. As different studies have conducted investigations on different sets of parameters in different operation ranges, there is expected variation in the results. This is because one of the parameters might be significant in a specified set of parameters and operation ranges, and it might be insignificant in another set of parameters and operation ranges. Apart from mechanical property optimization-based studies [166], the statistical-based models can also be used for weld quality assessment [167] and monitoring, and early prediction of weld defects.

**Figure 6:**
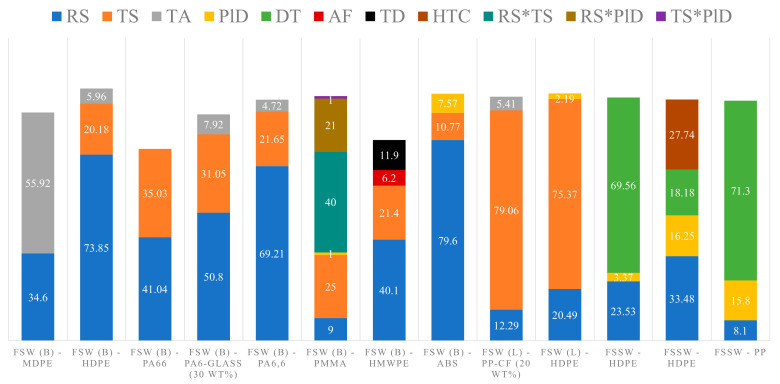
Percentage contribution of parameters for joint strength (tensile) of butt-joint FSW (MDPE [95], HDPE [1], PA66 [99], PMMA [122], HMWPE [168], Glass-filled PA6 (30 wt.%) [104], PA6,6 [113], ABS [43]), lap-joint FSW (HDPE [151], PP-CF20 [148]), and FSSW (HDPE [157,165], PP [158]). AF = Axial Force, DT = Dwell Time, HTC = Heat Transfer Coefficient, PlD = Plunge Depth, RS = Rotational Speed, TA = Tilt Angle, TD = Tool Diameter, TS = Traverse Speed. (B) = Butt-Joint Configuration, (L) = Lap-Joint Configuration. ABS = Acrylonitrile Butadiene Styrene, CF = Carbon Fiber, HDPE = High-Density Polyethylene, HMWPE = High Molecular Weight Polyethylene, MDPE = Medium-Density Polyethylene, PA = Polyamide (Nylon), PMMA = Polymethyl Methacrylate, PP = Polypropylene.

The ANOVA results of all the studies are summarized in Table 8. Several thermoplastic materials have been investigated for butt-joint FSW, which include HDPE [1], Nylon-66 [99], glass-filled Nylon 6 (30 wt.%) [104], HMWPE (Teflon stationary shoulder tool with threaded pin) [168], polyamide-6,6 (tapered cylindrical pin) [113], and ABS (3D-printed, wooden stationary shoulder) [43]. All these studies have suggested that rotational speed is the first and traverse speed is the second most influential parameters that affect joint strength. The total contribution of these two parameters on joint strength have been reported from 61.50% to 94.03%, with the contribution of rotational speed from 40.10% to 79.60% and traverse speed from 10.77% to 35.03%. Also, it has been reported that rotational speed is the most significant parameter, followed by traverse speed for impact strength [99] and percent elongation [104]. Adibeig et al. [122] have studied PMMA using a double-step shoulder tool with threaded pin. They have identified that the combination of rotational and traverse speeds most significantly affects joint tensile strength, followed by traverse speed, combination of rotational speed and pin diameter, and rotational speed as other significant factors. Saeedy and Givi [95] have studied MDPE. For tensile strength, they have reported that the tilt angle is the most significant parameter followed by the rotational speed; while for percent elongation, they have reported the rotational speed as the most significant parameter followed by tilt angle. Moochani et al. [129] have studied polypropylene using a heat-assisted stationary shoulder tool. For tensile strength, they have reported that the tool temperature is the most significant parameter followed by traverse and rotational speeds; while for percent elongation, rotational speed is the most significant parameter followed by traverse speed and tool temperature. Azarsa and Mostafapour [11] have studied HDPE sheets using a heat-assisted and PTFE-coated stationary shoulder tool with threaded pin. The reported ranking order for the flexural strength is: traverse speed, rotational speed, and combination of sheet temperature and traverse speed. Most of the above butt-joint configuration studies have also been summarized in columns 1 to 8 of Figure 6, which shows that most of the studies suggest that rotational and traverse speeds are the most important parameters that affect joint tensile strength and other mechanical properties. Some other parameters which have been identified to significantly affect (>5% contribution) the joint tensile strength are: axial force, tool diameter, tilt angle, and tool temperature. Apart from sheets, Mosavvar et al. [127] have studied butt-joint FSW of HDPE pipes using the threaded cylindrical pin. The reported ranking order for the yield strength is: rotational speed, tool offset (used as tilt angle in FSW of sheets), and traverse speed.

Very limited statistical studies are available on the FSW of thermoplastic sheets in lap-joint configuration. Ahmadi et al. [148] have investigated PP-carbon fiber (20 wt.%) composite with grooved cylindrical-conical pin. Yan et al. [151] have studied underwater FSW of HDPE using a double-pin tool. Regardless of the type of thermoplastic material, tool geometry, and medium configurations, both studies have reported traverse speed as the most significant parameter, and rotational speed as the second most significant parameter. The contribution of these two parameters on joint strength is reported to be around 91.35–95.86%, as can be seen in columns 9 and 10 of Figure 6. This means that optimizing mainly just these two parameters can help with achieving the maximum joint strength for given tooling and medium configurations. Also, optimizing just these two variables along with variations of tooling and medium configurations can help with the selection of best possible conditions for achieving maximum joint strength.

Limited statistical studies are available on the FSSW of thermoplastic sheets. Bilici et al. [157] have studied HDPE, and have reported dwell time as the most significant parameter for joint strength, followed by rotational speed and plunge depth. In another study, Bilici [158] have studied polypropylene, and have suggested dwell time as the most significant parameter for joint strength, followed by plunge depth and rotational speed. Lambiase et al. [155] have studied polycarbonate, and have shown dwell time as the most significant parameter for joint strength, followed by plunge rate, waiting time, and combination of plunge rate and dwell time. Bilici et al. [165] have studied HDPE with varying tool materials to investigate the effects of different tool materials and their heat transfer coefficients on the joint strength. They have reported rotational speed as the most significant parameter for joint strength, followed by heat transfer coefficient of tool material, dwell time, and plunge depth. Stan et al. [169] have studied the FSSW of HDPE-MWCNT (Multi-Walled Carbon Nanotubes) composite, and for joint quality, they have suggested the ranking order: dwell time > rotational speed > plunge depth > MNWCT wt.%. Most of the studies suggest that dwell time is the most significant parameter for joint strength, while other significant parameters (>5% contribution) that have been reported are rotational speed, plunge depth, and heat transfer coefficient of the tool material. These results are also summarized in the form of columns 11 to 13 of Figure 6.

For optimization of FSSW of thermoplastics polymers, the machine learning techniques like artificial neural networks (ANN) have also been utilized. Kurtulmuş and Kiraz [170] have developed the ANN model for HDPE sheets (dataset of 64 welding cases). The input dataset included the rotational speed, stirring time, and plunge depth, while the output of the model was lap-shear fracture load of the weld joint. Also, Chavan and Shete [171] have developed the ANN model for parametric analysis of HDPE sheets. The ANN models have also been developed for polycarbonate sheets [155,172], to optimize the welding conditions. The ANN-based models can also be used for mechanical behavior modeling [173], prediction of mechanical properties [174], and early prediction of weld defects.

## 6. Techno-Economic Aspects

FSW is a greener welding technique because of its low power consumption with no harmful welding byproducts, like greenhouse gases and toxic fumes. This technique can help automotive and other industries to cut down on their carbon footprint from their manufacturing chains. Apart from automotive industries, it has broad applications in other structural industries, like aerospace and marine industries. It is a unique welding technique that is widely popular among both metallic-based and polymeric-based industries. It has not only successfully joined similar and dissimilar metal and polymer parts but has also successfully developed polymer-metallic hybrid joints. FSW has the potential to compete and excel other metallic-based and polymeric-based welding techniques. It requires no preparation of parts, less processing time, no consumables, low-cost equipment, ability to join difficult to weld materials, and simple processing steps which can be easily automated for mass manufacturing. The technique is already in use in major car industries like Mazda and aerospace industries like Boeing. However, the large-scale, cost-effective, robotic FSW could be the answer to the next generation of flexible manufacturing in automotive, shipbuilding, and aerospace industries.

For thermoplastic polymers and their composites, the FSW technique is still in the development phase because most of the research is focused on butt- and lap-joint configurations. There is a serious need to transfer knowledge to other types of joints (like T-joint), and especially pipe and tube welding, which will create widespread applications and can also greatly help to cater to the already established piping, tubing, and tank construction industries. Also, apart from a few studies which have been focused on the development of specialized automated and robotic platforms to perform FSW of thermoplastics, most of them are focused towards using milling machines with fixtures. Further research into the development of low-cost, lightweight, and specialized platforms can also create extensive applications for the process because of the portability to use in field areas.

## 7. Concluding Remarks

The literature available on the FSW and FSSW of thermoplastic polymers and polymer composites is vast and has been classified into several classes in this study. The main classifications include the thermoplastic materials used (similar and dissimilar thermoplastic polymers and polymer composites), joint configuration (butt-, lap-, T-joint, etc.), tooling conditions (machine, tool type, tool material), medium conditions (submerged, non-submerged), and study types (parametric, phenomenological, tool design). Further, a detailed review focusing on the FSW and FSSW between similar thermoplastic polymers and polymer composites has been carried out. The effect of different process parameters, tooling, and medium conditions on the mechanical properties of the weld joint were covered in detail.

For the butt-joint, general guidelines have been developed that suggest that the double-side cooling-assisted (weld nugget cooling) FSW technique should be carried out using PTFE-coated and heat-assisted stationary shoulder tool. The welding sheets should be pre-heated, and the tool pin should be either a square pin, convex pin, tapered pin with groove, or threaded-tapered pin with a chamfer. These guidelines ensure a butt-joint weld with superior mechanical properties and less defects.

There are few studies available on the FSW of thermoplastics in lap configuration. However, the developed guidelines suggest that the better joint mechanical properties are achieved under submerged conditions. Also, the threaded cylindrical-conical pin and cone frustum pin profiles should be used for better weld joint strength.

There are also limited studies available on FSSW of thermoplastic polymers and polymer composites. The general guidelines suggest that welding should be performed with longer dwell times using tools made of pure copper material with tapered cylindrical and tapered threaded pin profiles. The available studies have also suggested that the plunge rate does not affect the weld joint strength, so it should not be investigated further in future parametric-based studies.

The statistical-based studies like ANOVA have helped with the determination of significant parameters for FSW and FSSW of thermoplastic polymers and polymer composites. For butt-joint configuration FSW, the most influential parameters are the rotational and traverse speeds that affect joint tensile strength and other mechanical properties. Other influential parameters are axial force, tool diameter, tilt angle, and tool temperature. For FSW of lap-joints, the most influential parameters are the traverse and rotational speeds, as their contribution on joint strength is reported to be around 91.35–95.86%. For FSSW, the most influential parameter for weld joint strength is the dwell time. Other influential parameters are rotational speed, plunge depth, and heat transfer coefficient of the tool material.

In a nutshell, the optimum welding conditions for FSW and FSSW of thermoplastic materials need to be determined for achieving high-quality joints with superior mechanical properties. The optimization of these welding conditions is a challenging task, as FSW and FSSW of thermoplastics depends on many factors. The available studies have only determined the local optimum conditions for the FSW of thermoplastics. There is a lack of detailed study for establishing the global optimal welding conditions. The developed general guidelines and the most influential parameters determined, and their operating ranges established from previous studies, can help with the development of a smart and extensive experimentation plan for future studies to determine the global optimum conditions.

There are two main areas related to FSW of thermoplastics with very few available studies. These two areas need to be studied in detail in the future to close the research gaps. Firstly, there are very few studies available on the FSW of thermoplastic pipes. In the future, the existing knowledge for thermoplastic sheets needs to be transferred for piping, tubing, and tank construction, as the thermoplastic-based materials have huge applications in these industrial areas. Secondly, there are also very few studies available that have focused on the development of a FE-based model for FSW of thermoplastic materials. Nowadays, the availability of low-cost yet powerful computational resources is paving the way for simulation and optimization of physical phenomena. The FE-based models are becoming widely popular as the cost is greatly reduced by performing less experimentations, required only for model verification purposes. The future studies should be focused on the development of comprehensive FE-based models of FSW that can help with maximizing the weld strength by the consideration of a greater number of process parameters along wide operating ranges. Such a model can lower the cost burden of large numbers of experimentations, as the model can be validated using a few experimental cases of the FSW of thermoplastics.

## Figures and Tables

**Table 1:** Research highlights of review articles and book chapters from the literature.

Reference.	Year	Article Type	Research Highlights
Pawar and Shete [36]	2013	Review	An early short review on FSW of Al and PE sheets, mostly using Taguchi and response surface methods-based investigations for optimization of welding process parameters.
Gao et al. [37]	2017	Review	A general review on friction stir welding and processing (weld tool, parameter optimization) of thermoplastic materials, and dissimilar FSW to form thermoplastic-metallic hybrid welds.
Eslami et al. [38]	2017	Review	A review on the different types of conventional and specialized welding tools for FSW and FSSW of thermoplastic materials.
Huang et al. [39]	2018	Review	A general review on FSW (parameters, tooling and medium conditions, thermo-mechanical behavior, defects) and processing (tooling and medium conditions) for thermoplastic materials, and dissimilar FSW to form thermoplastic-metallic hybrid welds.
Kumar et al. [40]	2018	Review	A general review (material compatibility, tool design, process parameters, material and heat distribution models, numerical modeling) related to the weldability and process capability of FSW of similar and dissimilar thermoplastics.
Haghshenas and Khodabakhshi [41]	2019	Review	A review on the FSW of aluminum-polymeric hybrid welds.
Mishra et al. [42]	2019	Book Chapter	A book chapter on polymers and joining of polymers using FSW and other techniques.
Singh et al. [43]	2020	Book Chapter	A book chapter on the FSW of three-dimensional (3D)-printed thermoplastics.
Zafar et al. [44]	2017	Overview	An overview on FSW of polymers.
Iftikhar et al. [45]	2020	Overview	An overview on FSW of HDPE.

**Table 2:** Butt-joint friction stir welding of thermoplastic polymers and polymer composites using the cylindrical pin profile tool.

Reference	Year	Material	Passes	Range	Conclusions/Optimum Conditions
Saeedy and Givi [95]	2010	MDPE	Single	RS = 1400–2000 rpm, TA = 1–2°, TS = 15 mm/min	RS = 1600 rpm, TA = 1°, TS = 15 mm/min for maximum tensile strength and % elongation
Saeedy and Givi [96]	2011	MDPE	Single	RS = 1000–1800 rpm, TS = 12–20 mm/min, TA = 1–2°	RS = 1400 rpm, TS = 12 mm/min, TA = 1° for highest tensile strength
Saeedy and Givi [97]	2011	HDPE	Single	RS = 1000–1600 rpm, TA = 1–3°, TS = 15 mm/min	Tensile strength is maximum at RS = 1400 rpm, while it decreases with increasing TA. Increasing RS or TA decreases % elongation
Bozkurt [1]	2012	HDPE	Single	RS = 1500–3000 rpm, TS = 45–115 mm/min, TA = 1–3°	RS = 3000 rpm, TS = 115 mm/min, TA = 3° for maximum tensile strength
Abdel-Gwad et al. [98]	2015	HDPE	Single	RS = 580–1800 rpm, TS = 14–48 mm/min, TA = 1°	RS = 930 rpm, TS = 25 mm/min, TA = 1° for maximum tensile strength, impact strength, and fatigue life
Husain et al. [99]	2015	PA66	Single	RS = 780–2000 rpm, TS = 27–62 mm/min	RS = 1570 rpm, TS = 42 for maximum tensile and impact strengths
Bilici et al. [100]	2017	HDPE	Single	PD = 4–6 mm, SD = 20–30 mm, RS = 600–1500 rpm, TS = 30–60 mm/min, TA = 1°, SA = 6°	RS = 900 rpm, TS = 45 mm/min, PD = 5 mm, SD = 25 mm, TA = 1°, SA = 6° for maximum tensile strength
Raouache et al. [101]	2018	HDPE	Single	RS = 500–2000 rpm, TS = 20–63 mm/min	RS = 710 rpm and TS = 20 mm/min, and RS = 1000 rpm and TS = 40 mm/min for maximum tensile strength
Sheikh-Ahmad et al. [102,103]	2018, 2019	HDPE-carbon black (2.3 wt.%)	Single	RS = 800–1200 rpm, TS = 20–40 mm/min, PlD = 4.1–4.2 mm	Plunge depth significantly affects tensile strength, strength increases significantly by increasing plunge depth
Kumar et al. [104]	2019	Glass-filled PA6 (30 wt.%)	Single	RS = 400–600 rpm, TS = 0.2–0.4 mm/s, TA = 0–2°, StD = 0.2 mm	RS = 600 rpm, TS = 0.2 mm/s, TA = 2° for maximum tensile strength and % elongation
Mishra et al. [42]	2019	HDPE	Single	RS = 500–800 rpm, TS = 10–30 mm/min, TA = 1°, PlD = 0.1 mm	RS = 800 rpm, TS = 10 mm/min, TA = 1°, PlD = 0.1 mm for maximum tensile strength
Singh et al. [43]	2020	ABS (3D Printed)	Single	RS = 900–1100 rpm, TS = 5–9 mm/min, PlD = 1.5–2.5 mm	RS = 1100 rpm, TS = 9 mm/min, PlD = 2 mm for maximum tensile strength
Arici and Sinmaz [92]	2005	MDPE	Double	RS = 600–1000 rpm, TS = 12.5–60 mm/min, TA = 0–1°	RS = 1000 rpm, TS = 12.5 mm/min, TA = 1° for maximum tensile strength (RS = 1000 rpm, TS = 25 mm/min, TA = 1° for overall tensile and bending properties)
Arici and Selale [93]	2007	MDPE	Double	TA = 0–5°, TS = 12.5–40 mm/min, RS = 1000 mm/min	TA = 1°, TS = 12.5 mm/min, RS = 1000 mm/min for maximum tensile strength
Saeedy and Givi [94]	2010	HDPE	Double	RS = 1200–1600 rpm, TS = 12 mm/min, TA = 1°	RS = 1400 rpm, TS = 12 mm/min, TA = 1° for maximum tensile strength and % elongation

PD = Pin Diameter, PlD = Plunge Depth, RS = Rotational Speed, SA = Shoulder Angle, SD = Shoulder Diameter, StD = Standoff Distance, TA = Tilt Angle, TS = Traverse Speed. HDPE = High-Density Polyethylene, MDPE = Medium-Density Polyethylene, PA = Polyamide (Nylon).

**Table 3:** Butt-joint FSW of thermoplastic polymer and polymer composite sheets with varying pin profiles.

Reference	Year	Material	Tool Type	Range	Optimum Conditions
Kiss and Czigány [115]	2007	PP	Two milling tools with 8 grooves at slopes of 15° and 45°	RS = 450–1800 rpm, TS = 20–63 mm/min	RS = 1800 rpm, TS = 20 mm/min, and 45° groove slope tool for maximum tensile strength. Weld seam shows lower ductility as compared to base material
Rezgui et al. [105]	2010	HDPE	Threaded cylindrical pin	RS = 900–1700 rpm, TS = 16–44 mm/min, HT = 9–20 s, PS = M10–M14	RS = 910 rpm, TS = 29 mm/min, HT = 9 s, PS = M12 for maximum flow stress; RS = 1700 rpm, TS = 44 mm/min, HT = 15 s, PS = M12 for maximum yield stress
Payganeh et al. [120]	2011	PP-GF30	Pin = triangular, threaded triangular, tapered with groove, cylindrical with groove	RS = 500 rpm, TS = 12 mm/min, TA = 1°	Tapered pin with groove provides maximum tensile strength
Rezgui et al. [106]	2011	HDPE	Threaded pin	RS = 653–1700 rpm, TS = 16–44 mm/min, TPS = 377–528 mm^2^	Effect of RS is insignificant for longitudinal flow stress. TS = 24 mm/min, TPS = 401 mm^2^ for maximum longitudinal flow stress
Inaniwa et al. [111]	2013	HDPE, PA6, PVC	Right-hand threaded pin with anticlockwise rotation	RS = 800–1240 rpm, TS = 15–45 mm/min for HDPE; RS = 380–500 rpm, TS = 40–50 mm/min for PA6; RS = 1600–1800 rpm, TS = 10–30 mm/min for PVC; PlD = 4.7 mm	Optimal conditions for maximum joint strength of HDPE (RS = 1240 rpm, TS = 15 mm/min), PA6 (RS = 440 rpm, TS = 40 mm/min), PVC (RS = 1800 rpm, TS = 10 mm/min)
Panneerselvam and Lenin [110]	2014	PA6	Left-handed threaded pin	RS = 1000 rpm, TS = 10 mm/min	Anticlockwise rotation provides better tensile strength, Shore–D hardness, Izod strength, and Charpy strength than clockwise rotation of tool
Pirizadeh et al. [119]	2014	ABS	Two-shoulder rotation-prevented tool (self-reacting tool); Pin = simple and convex form	RS = 400–800 rpm, TS = 20–60 mm/min	RS = 400 rpm, TS = 40 mm/min, Pin = convex for maximum tensile strength
Shazly et al. [109]	2014	PC	Threaded pin	RS = 1000–1850 rpm, TS = 20–40 mm/min, TA = 1–3°	RS = 1220 rpm, TS = 40 mm/min, TA = 1° for maximum tensile strength
Hoseinlaghab et al. [2]	2015	HDPE	Pin = cylindrical, conical	SD = 10–20 mm, SL = 20 mm, PD = 5–8 mm, PL = 5–7 mm, RS = 900–1400 rpm, TS = 12.5–31.5 mm/min, TA = 0–2°	Cylindrical pin achieved maximum creep-resistant welds even more compared to parent material (SD = 20 mm, SL = 20 mm, PD = 6 mm, PL = 7 mm) with RS = 1120 rpm, TS = 31.5 mm/min, TA = 0°
Sadeghian and Givi [117]	2015	ABS	Pin = cylindrical and conical	RS = 900–1800 rpm, TS = 6–25 mm/min, TA = 0–2°, SD = 10–20 mm, PD = 5–8 mm	Pin = conical, TA = 2°, RS = 900 rpm, DR = 20/6, TS = 25 mm/min for maximum tensile strength
Zafar et al. [125,126]	2015, 2016	PA6	Double shoulder tool with threaded pin	RS = 300–1000 rpm, TS = 25 mm/min, DT = 15 s	Double shoulder tool is designed to control molten Nylon–6 flow as it has low viscosity. Visual inspection and microscopic analysis of weld zone suggests that 300 rpm (low speeds) rotational speed provides defect-free weld joint
Zafar et al. [108]	2016	PA6	Threaded pin	RS = 300–1000 rpm, TA = 0–3°, DT = 15 s, TS = 25 mm/min	RS = 300 rpm, TA = 0°, DT = 15 s, TS = 25 mm/min for maximum tensile strength
Kordestani et al. [121]	2017	PP-CF30, PP-GF30	Pin = square, threaded-tapered, threaded-tapered with a chamfer, four-flute threaded	RS = 2000 rpm, TS = 8 mm/min, TA = 5°	Maximum joint tensile and Izod impact strengths are provided by threaded-tapered pin with a chamfer
Adibeig et al. [122]	2018	PMMA	Double-step shoulder tool with threaded pin	RS = 250–500 rpm, TS = 16–20 mm/min, PlD = 3–3.5 mm/min	RS = 250 rpm, TS = 16 mm/min, PlD = 3.5 mm for maximum tensile strength
Derazkola and Simchi [112]	2018	PMMA	Frustum pin	RS = 1250–1600 rpm, TS = 25–50 mm/min, PlD = 0.2–0.4, TA = 0–4°	RS = 1600 rpm, TS = 25 mm/min, TA = 2°, PlD = 0.2 mm for maximum tensile strength
Moreno-Moreno et al. [107]	2018	HDPE	Stationary shoulder tool with threaded cylindrical pin	RS = 846–1036 rpm, TS = 14–25 mm/min, TA = 0°	RS = 1036 rpm, TS = 14 mm/min, TA = 0° for maximum tensile strength
Sahu et al. [118]	2018	PP	Pin = square and cylindrical	RS = 500–1000 rpm, TS = 5–25 mm/min, TA = 1°	Pin = square, RS = 750 rpm, TS = 15 mm/min, TA = 1° for maximum tensile strength
Kaddour et al. [116]	2019	HDPE	Pin = cylindrical, conical	RS = 720–1750 rpm, TS = 24–40 mm/min	Cylindrical pin, RS = 875 rpm, TS = 24 mm/min for maximum tensile strength
Nandhini et al. [113]	2019	PA6,6	Tapered cylindrical pin	RS = 1100–1500 rpm, TS = 10–20 mm/min, TA = 0–2°	RS = 1300 rpm, TS = 15 mm/min, TA = 2° for maximum tensile strength
Vakili-Tahami et al. [123]	2019	PMMA	Two-shoulder tool with threaded pin	RS = 250–500 rpm, TS = 16–20 mm/min, PlD = 3–3.5 mm	RS = 250 rpm, TS = 16 mm/min, PlD = 3.5 mm for maximum tensile strength and creep strength
Meyer et al. [114]	2019	PA6-GF30	Stationary shoulder tool with a threaded conical pin	SD = 20–24 mm, TA = 1–2°, AF = 1500–2000 N, TS = 10–40 mm/min, RS = 2000 rpm	SD = 20 mm, RS = 2000 rpm, TA = 2°, AF = 2000 N, TS = 25 mm/min for maximum tensile strength
Mosavvar et al. [127]	2019	HDPE pipes	Threaded cylindrical pin	RS = 1500–2500 rpm, TS = 110–150 mm/min, TO = 2.5–4.5 mm	RS = 2500 rpm, TS = 110 mm/min, TO = 3.5 mm for maximum yield strength

AF = Axial Force, DR = Diameters Ratio, DT = Dwell Time, HT = Hold Time, PD = Pin Diameter, PL = Pin Length, PlD = Plunge Depth, PS = Pin Size, RP = Revolution Pitch, RS = Rotational Speed, SD = Shoulder Diameter, SL = Shoulder Length, TA = Tilt Angle, TS = Traverse Speed, TPS = Tool Plunged Surface, TO = Tool Offset. ABS = Acrylonitrile Butadiene Styrene, CF = Carbon Fiber, GF = Glass Fiber, HDPE = High-Density Polyethylene, PA = Polyamide (Nylon), PC = Polycarbonate, PMMA = Polymethyl Methacrylate, PP = Polypropylene, PVC = Polyvinyl Chloride.

**Table 4:** Heat-assisted butt-joint friction stir welding of thermoplastic polymers and polymer composites.

Reference	Year	Material	Tool Type	Range	Optimum Conditions
Aydin [130]	2010	UHMWPE	Pre-heated sheets, threaded pin	RS = 960–1960 rpm, TS = 10–20 mm/min, StT = room temp–80 °C	RS = 960 rpm, TS = 20 mm/min, StT = 50 °C for maximum tensile strength
Azarsa et al. [136]	2012	HDPE	Heat-assisted PTFE-coated stationary shoulder tool with threaded pin	RS = 1000–1600 rpm, ShT = 80–140 °C, TS = 10–40 mm/min	Coating reduces sticking of polymer melt. Weld defects and residual stress concentration are reduced, and joint strength is improved
Mostafapour and Azarsa [137]	2012	HDPE	Heat-assisted PTFE-coated stationary shoulder tool with threaded pin	RS = 1000–1600 rpm, TS = 10–40 mm/min, TT = 80–140 °C	RS = 1600 rpm, TS = 25 mm/min, TT = 140 °C for maximum tensile strength
Bagheri et al. [128]	2013	ABS	Fixed heated shoe tool with threaded pin	RS = 800–1600 rpm, TS = 20–80 mm/min, TT = 50–100 °C	RS = 1600 rpm, TS = 20 mm/min, TT = 100 °C for maximum tensile strength
Azarsa and Mostafapour [11]	2014	HDPE	Heat-assisted PTFE-coated stationary shoulder tool with threaded pin	RS = 710–1400 rpm, TS = 25–100 mm/min, TT = 70–150 °C	TS = 25 mm/min, RS = 1400 rpm, TT = 100 °C for maximum flexural strength
Mendes et al. [139]	2014	ABS	Robotic, heat-assisted tool with stationary shoulder, conical threaded pin	RS = 1000–1500 rpm, TS = 50–200 mm/min, AF = 1–2 kN, TT = 115 °C	RS = 1500 rpm, TS = 200 mm/min, AF = 2 kN for maximum tensile strength; RS = 1500 rpm, TS = 100 mm/min, AF = 1.5 kN for best strain
Vijendra and Sharma [140]	2015	HDPE	Induction-heat assisted tool with taper-threaded pin	RS = 1000–3000 rpm, TT = Room Temp.—55, TS = 50 mm/min	RS = 2000 rpm, TT = 45 °C, TS = 50 mm/min for maximum tensile strength
Mostafapour and Asad [138]	2016	PA6	Heat-assisted tool with stationary PTFE-coated shoe	RS = 500–800 rpm, TS = 20–30 mm/min, TT = 100–150 °C	RS = 730 rpm, TS = 20 mm/min, TT = 150 °C for maximum tensile strength
Nateghi and Hosseinzadeh [142]	2016	HDPE	Cooling-assisted (weld nugget cooling with CO_2_ gas), threaded pin tool	RS = 100–2200 rpm, TS = 40–80 mm/min, SD = 18–22 mm, CP = 0–2 bar	RS = 2200 rpm, TS = 40 mm/min, SD = 22 mm, CP = 2 bar for maximum tensile strength; RS = 1000 rpm, TS = 80 mm/min, SD = 18 mm, CP = 2 bar for minimum angular distortion
Banjare et al. [131]	2017	PP	Heat-assisted tool with threaded cylindrical pin	RS = 360–840 rpm, TS = 20–30 mm/min, TA = 2°, TPT = 110 °C (for experiments with heat-assisted FSW)	Heat-assisted tooling provides better joint mechanical properties (tensile strength, elongation) compared to non–heated tooling conditions
Moochani et al. [129]	2019	PP	Heat-assisted stationary shoulder	TT = 130–170 °C, RS = 360–950 rpm, TS = 24–60 mm/min	RS = 950 rpm, TS = 40 mm/min, and TT = 150 °C for high tensile strength and elongation
Nath et al. [141]	2019	PP	Self-heated tool with right-hand threaded cylindrical pin	TA = 1°, RS = 1600 rpm CW, TS = 0.1–0.3 mm/s, PlD = 0.1 mm, TPT = 373.15 K	TS = 0.3 mm/s for maximum tensile strength and % elongation
Laieghi et al. [132]	2020	PA6/NBR20—HNT	Heat-assisted tool with stationary PTFE-coated shoulder and threaded pin	RS = 900–1400 rpm, TS = 14 mm/min, HNT content = 3–7 wt.%, PlD = 0.9 mm, TT = 140 °C	RS = 900 rpm, TS = 14 mm/min, HNT content = 5%, PlD = 0.9 mm, TT = 140 °C for highest mechanical properties (tensile strength, hardness, impact strength)

AF = Axial Force, CP = Cooling Pressure, PlD = Plunge Depth, RS = Rotational Speed, SD = Shoulder Diameter, StT = Sheet Temperature, TA = Tilt Angle, TS = Traverse Speed, TPT = Tool Pre-heated Temperature, TT = Tool Temperature. ABS = Acrylonitrile Butadiene Styrene, HDPE = High-Density Polyethylene, HNT = Halloysite Nantotubes, NBR = Nitrile Butadiene Rubber, PA6 = Polyamide-6 (Nylon-6), PP = Polypropylene, UHMWPE = Ultra-High Molecular Weight Polyethylene.

**Table 5:** Range and average of the optimal rotational and traverse speeds for thermoplastic polymer and polymer composite sheets.

Reference	Material	Optimal Rotation Speed Range	Optimal Traverse Speed Range	Average Optimal Rotational Speed	Average Optimal Traverse Speed
[43]	ABS (I)	1100 rpm	9 mm/min	1100 rpm	9 mm/min
[117,119]	ABS (II)	400–900 rpm	25–40 mm/min	650 rpm	32.5 mm/min
[128,139]	ABS (III)	1500–1600 rpm	20–200 mm/min	1550 rpm	110.0 mm/min
[104]	Glass-filled PA6 (30 wt.%) (I)	600 rpm	0.2 mm/s	600 rpm	0.2 mm/s
[42,94,97,98,100,101]	HDPE (I)	710–1400 rpm	10–40 mm/min	1020 rpm	23.9 mm/min
[107,111,116]	HDPE (II)	875–1240 rpm	14–24 mm/min	1050 rpm	17.7 mm/min
[137,140]	HDPE (III)	1600–2000 rpm	25–50 mm/min	1800 rpm	37.5 mm/min
[92,93,95,96]	MDPE (I)	1000–1600 rpm	12–15 mm/min	1250 rpm	13.0 mm/min
[108,111]	PA6 (II)	300–440 rpm	25–40 mm/min	370 rpm	32.5 mm/min
[138]	PA6 (III)	730 rpm	20 mm/min	730 rpm	20.0 mm/min
[99]	PA66 (I)	1570 rpm	42 mm/min	1570 rpm	42.0 mm/min
[113]	PA6,6 (II)	1300 rpm	15 mm/min	1300 rpm	15.0 mm/min
[114]	PA6-GF30 (II)	2000 rpm	25 mm/min	2000 rpm	25.0 mm/min
[132]	PA6/NBR20-HNT (III)	900 rpm	14 mm/min	900 rpm	14.0 mm/min
[109]	PC (II)	1220 rpm	40 mm/min	1220 rpm	40.0 mm/min
[112,122,123]	PMMA (II)	250–1600 rpm	16–25 mm/min	700 rpm	19.0 mm/min
[115,118]	PP (II)	750–1800 rpm	15–20 mm/min	1275 rpm	17.5 mm/min
[129,141]	PP (III)	950–1600 rpm	0.3–40 mm/min	1275 rpm	20.2 mm/min
[111]	PVC (II)	1800 rpm	10 mm/min	1800 rpm	10.0 mm/min
[130]	UHMWPE (III)	960 rpm	20 mm/min	960 rpm	20.0 mm/min

I denotes FSW (single- and double-side) using cylindrical pin profile, II denotes FSW with other than cylindrical pin profile, and III denotes heat-assisted FSW.

**Table 6:** Lap-joint friction stir welding of thermoplastic polymers and polymer composites sheets.

Reference	Year	Material	Tool Type	Medium	Range	Optimum Conditions
Ahmadi et al. [147]	2012	PP-CF20	Pin = threaded cylindrical, threaded conical, simple cylindrical-conical, threaded cylindrical-conical	Non-submerged	RS = 1000 rpm, TS = 16 mm/min, TA = 1°	Threaded cylindrical-conical pin provides maximum joint strength
Ahmadi et al. [148]	2014	PP-CF20	Grooved cylindrical-conical pin	Non-submerged	RS = 800–1250 rpm, TS = 16–25 mm/min, TA = 0–2°	RS = 1250 rpm, TS = 25 mm/min, TA = 1° for maximum tensile shear strength
Gao et al. [150]	2014	HDPE	Threaded pin	Submerged	PlD = 0.4 mm, TA = 0°, RS = 1200–2400 rpm, TS = 30–60 mm/min	RS = 1800 rpm, TS = 45 mm/min, PlD = 0.4 mm, TA = 0° for maximum joint strength
Yan et al. [151]	2017	HDPE	Double-pin tool	Submerged	RS = 700–1300 rpm, TS = 20–40 mm/min, PlD = 0–0.2 mm	TS = 20 mm/min, RS = 1300 rpm, PlD = 0.1 mm for maximum joint strength
Derazkola and Simchi [149]	2018	PMMA	Pin = cone, square, and triangle frustums	Non-submerged	RS = 810–1920 rpm, TS = 25–50 mm/min, TA = 2°, PlD = 0.2 mm	Pin = cone frustum, RS = 1600 rpm, TS = 25 mm/min, TA = 2°, PlD = 0.2 mm for maximum joint strength

PlD = Plunge Depth, RS = Rotational Speed, TA = Tilt Angle, TS = Traverse Speed. CF = Carbon Fiber, HDPE = High-Density Polyethylene, PMMA = Polymethyl Methacrylate, PP = Polypropylene.

**Table 7:** Friction stir spot welding of thermoplastic polymer and polymer composite sheets.

Reference	Year	Material	Tool Type	Range	Optimum Conditions
Bilici et al. [157]	2011	HDPE	Tapered cylindrical pin	RS = 700–1100 rpm, DT = 20–60 s, PlD = 5.7–6.7 mm	DT = 60 s, RS = 700 rpm, PlD = 6.2 mm for maximum joint strength
Bilici [158]	2012	PP	Tapered cylindrical pin	DT = 50–150 s, PlD = 5.7–6.7 mm, RS = 700–1100 rpm	DT = 100 s, PlD = 5.7 mm, RS = 900 rpm for maximum joint strength
Bilici [163]	2012	PP	Pin = straight cylindrical, threaded cylindrical, tapered cylindrical, square	RS = 900 rpm, DT = 105 s, DeT = 50 s, PR = 0.33 mm/s, PlD = 0.20 mm	Tapered cylindrical pin for maximum joint strength
Bilici and Yukler [13]	2012	HDPE	Tapered cylindrical pin	RS = 280–1400 rpm, PR = 3.3–20.8 mm/s, PlD = 5.5–7.0 mm, DT = 8–90 s	RS = 710 rpm (conditions of DT = 50 s, PR = 3.3 mm/s, PlD = 6 mm) for maximum joint strength
Bilici and Yükler [164]	2012	HDPE	Pin = straight cylindrical, tapered cylindrical, threaded cylindrical, triangular, square, hexagonal	RS = 560–1120 rpm, DT = 8–90 s, DeT = 0–60 s, PlD = 0.2–1.2 mm	RS = 710 rpm, PlD = 5.7 mm, DT = 45 s, DeT = 30 s for maximum joint strength
Kurtulmus [159]	2012	PP	Tapered cylindrical pin	DT = 30–150 s, PlD = 5.6–7 mm, RS = 560–1400 rpm, PR = 0.25–3.55 mm/min	PR has negligible effect on joint strength; RS = 900 rpm, PlD = 5.7 mm, DT = 120 s for maximum joint strength
Lambiase et al. [155]	2015	PC	Cylindrical pin	PR = 8–46 mm/min, RS = 1500–5400 rpm, DT = 0–20 s, WT = 20 s, PHT = 20 s, PlD = 4.4 mm	PR = 8 mm/min, DT = 20 s, RS = 1500 rpm for maximum joint strength
Tasdemir et al. [160]	2016	HDPE-glass hollow spheres	Tapered cylindrical pin	Glass sphere = 0–20 wt.%, RS = 560–1400 rpm, DT = 15–75 s, Det = 0–60 s, PR = 0.33 mm/s, PlD = 0.2 mm	Joint strength is maximum at GS = 10 wt.% and RS = 1120 rpm, at GS = 20 wt.% and DT = 60 s, and at GS = 20 wt.% and DeT = 30 s
Lambiase et al. [156]	2017	PC	Cylindrical pin	SD = 10–20 mm, PlP = 0.8–1.6 MPa, PR = 8 mm/min, SrT = 15 s, CT = 15 s, RS = 1260 rpm	SD = 10 mm, PlP = 1.2 MPa for maximum joint strength
Bilici et al. [165]	2018	HDPE	Tool material = copper, aluminum 1050, SAE 1020 steel, 316 stainless-steel	RS = 560–1120 rpm, DT = 30–75 s, PlD = 0.1–0.4 mm, HTC = 16–385 W/m.K	RS = 710 rpm, tool material = copper (HTC = 385 W/m.K), DT = 45 s, PlD = 0.2 mm for maximum joint strength
Yan et al. [161]	2018	ABS	Triflute pin	RS = 400–1000 rpm, PlD = 0–0.6 mm, DT = 1–51 s, PR = 15 mm/min, WT = 25 s	Triflute pin provides better joint strength than a cylindrical pin

CT = Cooling Time, DeT = Delay Time, DT = Dwell Time, HTC = Heat Transfer Coefficient, PHT = Pre-Heating Time, PlD = Plunge Depth, PlP = Plunging Pressure, PR = Plunge Rate, RS = Rotational Speed, SD = Shoulder Diameter, SrT = Stirring Time, WT = Waiting Time. ABS = Acrylonitrile Butadiene Styrene, GS = Glass Spheres, HDPE = High-Density Polyethylene, PC = Polycarbonate, PP = Polypropylene.

**Table 8:** Analysis of variance (ANOVA)-based studies of friction stir welding of thermoplastic polymer and polymer composite sheets.

Reference	Year	Material	Technique/Tool Type	Range	ANOVA Results
Saeedy and Givi [95]	2010	MDPE	Butt-joint FSW	RS = 1400–2000 rpm, TA = 1–2°, TS = 15 mm/min	RS (63.98%) > TA (32.88%) for % elongation; TA (55.92%) > RS (34.60%) for tensile strength
Bozkurt [1]	2012	HDPE	Butt-joint FSW	RS = 1500–3000 rpm, TS = 45–115 mm/min, TA = 1–3°	RS (73.85%) > TS (20.18%) > TA (5.96%) for tensile strength
Azarsa and Mostafapour [11]	2014	HDPE	Butt-joint FSW, heat-assisted and PTFE-coated stationary shoulder tool with threaded pin	RS = 710–1400 rpm, TS = 25–100 mm/min, TT = 70–150 °C	TS > RS > TS + TT for flexural strength
Husain et al. [99]	2015	PA66	Butt-joint FSW	RS = 780–2000 rpm, TS = 27–62 mm/min	RS (41.04%) > TS (35.03%) for tensile strength; RS (55.98%) > TS (31.75%) for impact strength
Adibeig et al. [122]	2018	PMMA	Butt-joint FSW, double-step shoulder tool with threaded pin	RS = 250–500 rpm, TS = 16–20 mm/min, PlD = 3–3.5 mm/min	RS × TS (40%), TS (25%), RS × PlD (21%), RS (9%), TS × PlD (1%), PlD (1%) for tensile strength
Eslami et al. [168]	2018	HMWPE	Butt-joint FSW, Teflon stationary shoulder tool with threaded pin	RS = 1500–2500 rpm, TS = 30–70 mm/min, AF = 800–1100 N, TD = 3–5 mm	RS (40.10%) > TS (21.4%) > TD (11.90%) > AF (6.20%) for joint strength
Kumar et al. [104]	2019	Glass-filled PA6 (30 wt.%)	Butt-joint FSW	RS = 400–600 rpm, TS = 0.2–0.4 mm/s, TA = 0–2°, StD = 0.2 mm	RS (50.80%) > TS (31.05%) > TA (7.92%) for tensile strength; RS (71.15%) > TS (26.27%) > TA (2.30%) for % elongation
Moochani et al. [129]	2019	PP	Butt-joint FSW, heat-assisted stationary shoulder	TT = 130–170 °C, RS = 360–950 rpm, TS = 24–60 mm/min	TT > TS > RS for tensile strength; RS > TS > TT for % elongation
Nandhini et al. [113]	2019	PA6,6	Butt-joint FSW, tapered cylindrical pin	RS = 1100–1500 rpm, TS = 10–20 mm/min, TA = 0–2°	RS (69.21%) > TS = 21.65% > TA = 4.72% for tensile strength
Mosavvar et al. [127]	2019	HDPE pipes	Butt-joint FSW, threaded cylindrical pin	RS = 1500–2500 rpm, TS = 110–150 mm/min, TO = 2.5–4.5 mm	RS (43.6%) > TO (30.3%) > TS (26.1%) for yield strength
Singh et al. [43]	2020	ABS (3D Printed)	Butt-joint FSW, wooden stationary shoulder	RS = 900–1100 rpm, TS = 5–9 mm/min, PlD = 1.5–2.5 mm	RS (79.60%) > TS (10.77%) > PlD (7.57%) for tensile strength
Ahmadi et al. [148]	2014	PP-CF (20 wt.%)	Lap-joint FSW, grooved cylindrical-conical pin	RS = 800–1250 rpm, TS = 16–25 mm/min, TA = 0–2°	TS (79.06%) > RS (12.29%) > TA (5.41%) for tensile shear strength
Yan et al. [151]	2017	HDPE	Lap-joint Submerged FSW, double-pin tool	RS = 700–1300 rpm, TS = 20–40 mm/min, PlD = 0–0.2 mm	TS (75.37%) > RS (20.49%) > PlD (2.19%) for joint strength
Bilici et al. [157]	2011	HDPE	FSSW, Tapered cylindrical pin	RS = 700–100 rpm, DT = 20–60 s, PlD = 5.7–6.7 mm	DT (69.56%) > RS (23.53%) > PlD (3.37%) for joint strength
Bilici [158]	2012	PP	FSSW, Tapered cylindrical pin	DT = 50–150 s, PlD = 5.7–6.7 mm, RS = 700–1100 rpm	DT (71.3%) > PlD (15.8%) > RS (8.1%) for joint strength
Lambiase et al. [155]	2015	PC	FSSW, Cylindrical pin	PR = 8–46 mm/min, RS = 1500–5400 rpm, PHT = 0–20 s, DT = 0–20 s, WT = 0–20 s, PlD = 4.4 mm	DT > PR > WT > PR × DT for joint strength
Bilici et al. [165]	2018	HDPE	FSSW, tool material = copper, aluminum 1050, SAE 1020 steel, 316 stainless steel	RS = 560–1120 rpm, DT = 30–75 s, PlD = 0.1–0.4 mm, HTC = 16–385 W/m.K	RS (33.48%) > HTC (27.74%) > DT (18.18%) > PlD (16.25%) for joint strength

AF = Axial Force, DT = Dwell Time, HTC = Heat Transfer Coefficient, PHT = Pre-Heating Time, PlD = Plunge Depth, PR = Plunge Rate, RS = Rotational Speed, StD = Standoff Distance, TA = Tilt Angle, TD = Tool Diameter, TO = Tool Offset, TS = Traverse Speed, TT = Tool Temperature, WT = Waiting Time. ABS = Acrylonitrile Butadiene Styrene, CF = Carbon Fiber, HDPE = High-Density Polyethylene, HMWPE = High Molecular Weight Polyethylene, MDPE = Medium-Density Polyethylene, PA = Polyamide (Nylon), PC = Polycarbonate, PMMA = Polymethyl Methacrylate, PP = Polypropylene.

## References

[1] Bozkurt Y. (2012). The optimization of friction stir welding process parameters to achieve maximum tensile strength in polyethylene sheets. Mater. Des..

[2] Hoseinlaghab S., Mirjavadi S.S., Sadeghian N., Jalili I., Azarbarmas M., Besharati Givi M.K. (2015). Influences of welding parameters on the quality and creep properties of friction stir welded polyethylene plates. Mater. Des..

[3] Sabry I., El-Kassas A.M., Mourad A.-H.I., Thekkuden D.T., Abu Qudeiri J. (2019). Friction Stir Welding of T-Joints: Experimental and Statistical Analysis. J. Manuf. Mater. Process..

[4] El-Kassas A.M., Sabry I., Mourad A.-H.I., Thekkuden D.T. (2019). Characteristics of potential sources-vertical force, torque and current on penetration depth for quality assessment in friction stir welding of AA 6061 pipes. Int. Rev. Aerosp. Eng..

[5] Thomas W.M., Nicholas E.D., Needham J.C., Murch M.G., Temple-Smith P., Dawes C.J. (1991). Friction Stir Butt Welding. Int. Pat. Appl. No. PCT/GB92/02203 GB Pat. Appl..

[6] Mourad A.-H.I., Allam M., El Domiaty A. Study on the mechanical behavior of aluminum alloy 5083 friction stir welded joint. Proceedings of the Pressure Vessels and Piping Conference.

[7] Lee W.B., Jung S.B. (2004). The joint properties of copper by friction stir welding. Mater. Lett..

[8] Lee W.B., Lee C.Y., Chang W.S., Yeon Y.M., Jung S.B. (2005). Microstructural investigation of friction stir welded pure titanium. Mater. Lett..

[9] Afrin N., Chen D.L., Cao X., Jahazi M. (2008). Microstructure and tensile properties of friction stir welded AZ31B magnesium alloy. Mater. Sci. Eng. A.

[10] Fujii H., Cui L., Tsuji N., Maeda M., Nakata K., Nogi K. (2006). Friction stir welding of carbon steels. Mater. Sci. Eng. A.

[11] Azarsa E., Mostafapour A. (2014). Experimental investigation on flexural behavior of friction stir welded high density polyethylene sheets. J. Manuf. Process..

[12] Nelson T.W., Sorenson C.D., Johns C.J. (2004). Friction stir welding of polymeric materials. U.S. Patent.

[13] Bilici M.K., Yukler A.I. (2012). Effects of welding parameters on friction stir spot welding of high density polyethylene sheets. Mater. Des..

[14] Mourad A.-H.I., Harib K.H., El-Domiaty A. Fracture behaviour of friction stir spot welded joint. Proceedings of the American Society of Mechanical Engineers, Pressure Vessels and Piping Division/K-PVP Conference.

[15] Merzoug M., Mazari M., Berrahal L., Imad A. (2010). Parametric studies of the process of friction spot stir welding of aluminium 6060-T5 alloys. Mater. Des..

[16] Jordon J.B., Horstemeyer M.F., Daniewicz S.R., Badarinarayan H., Grantham J. (2010). Fatigue characterization and modeling of friction stir spot welds in magnesium AZ31 alloy. J. Eng. Mater. Technol..

[17] Yin Y.H., Sun N., North T.H., Hu S.S. (2010). Influence of tool design on mechanical properties of AZ31 friction stir spot welds. Sci. Technol. Weld. Join..

[18] Khan M.I., Kuntz M.L., Su P., Gerlich A., North T., Zhou Y. (2007). Resistance and friction stir spot welding of DP600: A comparative study. Sci. Technol. Weld. Join..

[19] Feng Z., Santella M.L., David S.A., Steel R.J., Packer S.M., Pan T., Kuo M., Bhatnagar R.S. (2005). Friction Stir Spot Welding of Advanced High-Strength Steels—A Feasibility Study. SAE Trans..

[20] Arici A., Mert Ş. (2008). Friction stir spot welding of polypropylene. J. Reinf. Plast. Compos..

[21] Paoletti A., Lambiase F., Di Ilio A. (2016). Analysis of forces and temperatures in friction spot stir welding of thermoplastic polymers. Int. J. Adv. Manuf. Technol..

[22] Kiss Z., Czigány T. (2012). Effect of welding parameters on the heat affected zone and the mechanical properties of friction stir welded poly(ethylene-terephthalate-glycol). J. Appl. Polym. Sci..

[23] Mendes N., Loureiro A., Martins C., Neto P., Pires J.N. (2014). Effect of friction stir welding parameters on morphology and strength of acrylonitrile butadiene styrene plate welds. Mater. Des..

[24] Mourad A.-H.I., Dehbi A. (2014). On use of trilayer low density polyethylene greenhouse cover as substitute for monolayer cover. Plast. Rubber Compos..

[25] Dehbi A., Mourad A.-H.I. (2016). Durability of mono-layer versus tri-layers LDPE films used as greenhouse cover: Comparative study. Arab. J. Chem..

[26] Fouad H., Mourad A.-H.I., Barton D.C. (2005). Effect of pre-heat treatment on the static and dynamic thermo-mechanical properties of ultra-high molecular weight polyethylene. Polym. Test..

[27] Yan Y., Shen Y., Zhang W., Guan W. (2017). Effects of friction stir spot welding parameters on morphology and mechanical property of modified cast nylon 6 joints produced by double-pin tool. Int. J. Adv. Manuf. Technol..

[28] Lambiase F., Paoletti A., Grossi V., Di Ilio A. (2019). Analysis of loads, temperatures and welds morphology in FSW of polycarbonate. J. Mater. Process. Technol..

[29] Jaiganesh V., Maruthu B., Gopinath E. (2014). Optimization of process parameters on friction stir welding of high density polypropylene plate. Procedia Eng..

[30] Panneerselvam K., Lenin K. (2012). Investigation on effect of tool forces and joint defects during FSW of polypropylene plate. Procedia Eng..

[31] Al Kuwaiti M.H., Mourad A.-H.I. Thermomechanical Characteristics of Compacted and Non-Compacted Plain Weave Woven Laminated Composites. Proceedings of the Pressure Vessels and Piping Conference.

[32] Al-Kuwaiti M.H.H., Mourad A.-H.I. (2015). Effect of different environmental conditions on the mechanical behavior of plain weave woven laminated composites. Procedia Eng..

[33] Czigány T., Kiss Z. Friction stir welding of fiber reinforced polymer composites. Proceedings of the International Conferences on Composite Materials.

[34] Strand S. Joining plastics-can friction stir welding compete?. Proceedings of the Electrical Insulation Conference and Electrical Manufacturing and Coil Winding Conference and Exhibition.

[35] Oliveira P.H.F., Amancio-Filho S.T., Dos Santos J.F., Hage E. (2010). Preliminary study on the feasibility of friction spot welding in PMMA. Mater. Lett..

[36] Pawar S.P., Shete M.T. (2013). Optimization of friction stir welding process parameter using taguchi method and response surface methodology: A review. Int. J. Res. Eng. Technol..

[37] Gao J., Cui X., Liu C., Shen Y. (2017). Application and exploration of friction stir welding/processing in plastics industry. Mater. Sci. Technol..

[38] Eslami S., Tavares P.J., Moreira P.M.G.P. (2017). Friction stir welding tooling for polymers: Review and prospects. Int. J. Adv. Manuf. Technol..

[39] Huang Y., Meng X., Xie Y., Wan L., Lv Z., Cao J., Feng J. (2018). Friction stir welding/processing of polymers and polymer matrix composites. Compos. Part A Appl. Sci. Manuf..

[40] Kumar R., Singh R., Ahuja I.P.S., Penna R., Feo L. (2018). Weldability of thermoplastic materials for friction stir welding—A state of art review and future applications. Compos. Part B Eng..

[41] Haghshenas M., Khodabakhshi F. (2019). Dissimilar friction-stir welding of aluminum and polymer: A review. Int. J. Adv. Manuf. Technol..

[42] Mishra D., Sahu S.K., Mahto R.P., Pal S.K., Pal K. (2019). Friction Stir Welding for Joining of Polymers. Strengthening and Joining by Plastic Deformation. Lecture Notes on Multidisciplinary Industrial Engineering.

[43] Singh S., Prakash C., Gupta M.K. (2020). On friction-stir welding of 3D printed thermoplastics. Materials Forming, Machining and Post Processing. Materials Forming, Machining and Tribology.

[44] Zafar A., Awang M., Khan S.R., Awang M. (2017). Friction stir welding of polymers: An overview. 2nd International Conference on Mechanical, Manufacturing and Process Plant Engineering. Lecture Notes in Mechanical Engineering.

[45] Iftikhar S.H., Mourad A.-H.I., Sheikh-Ahmad J. An overview of friction stir welding of high-density polyethylene. Proceedings of the Advances in Science and Engineering Technology International Conferences.

[46] Raza K., Shamir M., Qureshi M.K.A., Shaikh A.S., Zain-ul-abdein M. (2018). On the friction stir welding, tool design optimization, and strain rate-dependent mechanical properties of HDPE–ceramic composite joints. J. Thermoplast. Compos. Mater..

[47] Azhiri R.B., Mehdizad Tekiyeh R., Zeynali E., Ahmadnia M., Javidpour F. (2018). Measurement and evaluation of joint properties in friction stir welding of ABS sheets reinforced by nanosilica addition. Measurement.

[48] Thomas Thekkuden D., Mourad A.-H.I., Sherif M.M. Response surface analysis of statistical features of voltage and current in a GMAW powersource on welding v-groove joints. Proceedings of the Advances in Science and Engineering Technology International Conferences.

[49] Mozumder M.S., Mourad A.-H.I., Mairpady A., Pervez H., Haque M.E. (2018). Effect of TiO_2_ nanofiller concentration on the mechanical, thermal and biological properties of HDPE/TiO2 nanocomposites. J. Mater. Eng. Perform..

[50] Huang Y., Meng X., Xie Y., Lv Z., Wan L., Cao J., Feng J. (2018). Friction spot welding of carbon fiber-reinforced polyetherimide laminate. Compos. Struct..

[51] Thekkuden D.T., Santhakumari A., Sumesh A., Mourad A.-H.I., Rameshkumar K. (2018). Instant detection of porosity in gas metal arc welding by using probability density distribution and control chart. Int. J. Adv. Manuf. Technol..

[52] Gonçalves J., Dos Santos J.F., Canto L.B., Amancio-Filho S.T. (2015). Friction spot welding of carbon fiber-reinforced polyamide 66 laminate. Mater. Lett..

[53] Sabry I., Mourad A.-H.I., Thekkuden D.T. (2020). Optimization of metal inert gas-welded aluminium 6061 pipe parameters using analysis of variance and grey relational analysis. SN Appl. Sci..

[54] Ahmed W.K., Mourad A.-H.I. (2013). Strengthening of misaligned welded pipes with outer circumferentially crack using FRP bandage finite element analysis. J. Mech. Eng. Technol..

[55] Mejia E.B., Mourad A.-H.I., Ba Faqer A.S., Halwish D.F., Al Hefeiti H.O., Al Kashadi S.M., Cherupurakal N., Mozumder M.S. Impact on HDPE Mechanical Properties and Morphology due to Processing. Proceedings of the Advances in Science and Engineering Technology International Conferences.

[56] Kumar R., Singh R., Ahuja I.P.S. (2019). Mechanical, thermal and micrographic investigations of friction stir welded: 3D printed melt flow compatible dissimilar thermoplastics. J. Manuf. Process..

[57] Derazkola H.A., Eyvazian A., Simchi A. (2020). Modeling and experimental validation of material flow during FSW of polycarbonate. Mater. Today Commun..

[58] Simoes F., Rodrigues D.M. (2014). Material flow and thermo-mechanical conditions during Friction Stir Welding of polymers: Literature review, experimental results and empirical analysis. Mater. Des..

[59] Kiss Z., Czigány T. (2012). Microscopic analysis of the morphology of seams in friction stir welded polypropylene. Express Polym. Lett..

[60] Dehbi A., Mourad A.-H.I., Bouaza A. (2012). Degradation assessment of LDPE multilayer films used as a greenhouse cover: Natural and artificial aging impacts. J. Appl. Polym. Sci..

[61] Dehbi A., Mourad A.-H.I., Djakhdane K., Hilal-Alnaqbi A. (2015). Degradation of thermomechanical performance and lifetime estimation of multilayer greenhouse polyethylene films under simulated climatic conditions. Polym. Eng. Sci..

[62] Kumar R., Singh R., Ahuja I.P.S. (2019). Friction stir welding of 3D printed melt flow compatible dissimilar thermoplastic composites. Proc. Inst. Mech. Eng. Part C J. Mech. Eng. Sci..

[63] Kumar R., Singh R., Ahuja I.P.S. (2019). Friction stir welding of ABS-15Al sheets by introducing compatible semi-consumable shoulder-less pin of PA6-50Al. Measurement.

[64] Kumar R., Singh R., Ahuja I.S. (2020). Joining of 3D printed dissimilar thermoplastics with consumable tool through friction stir spot welding: A case study. Encyclopedia of Renewable and Sustainable Materials.

[65] Sheikh-Ahmad J.Y., Ali D., Meng F. (2018). Design and Implementation of a Force Dynamometer for Friction Stir Welding. Arab. J. Sci. Eng..

[66] Eslami S., Mourão L., Viriato N., Tavares P.J., Moreira P.M.G.P. (2018). Multi-axis force measurements of polymer friction stir welding. J. Mater. Process. Technol..

[67] Mendes N., Neto P., Simão M.A., Loureiro A., Pires J.N. (2016). A novel friction stir welding robotic platform: Welding polymeric materials. Int. J. Adv. Manuf. Technol..

[68] Rezaee Hajideh M., Farahani M., Alavi S.A.D., Molla Ramezani N. (2017). Investigation on the effects of tool geometry on the microstructure and the mechanical properties of dissimilar friction stir welded polyethylene and polypropylene sheets. J. Manuf. Process..

[69] Kumar S., Roy B.S. (2019). Novel study of joining of acrylonitrile butadiene styrene and polycarbonate plate by using friction stir welding with double-step shoulder. J. Manuf. Process..

[70] Gao J., Shen Y., Xu H. (2016). Investigations for the mechanical, macro-, and microstructural analyses of dissimilar submerged friction stir welding of acrylonitrile butadiene styrene and polycarbonate sheets. Proc. Inst. Mech. Eng. Part B J. Eng. Manuf..

[71] Eslami S., Tavares P.J., Moreira P.M.G.P. (2017). Fatigue life assessment of friction stir welded dissimilar polymers. Procedia Struct. Integr..

[72] Eslami S., Farahani B.V., Tavares P.J., Moreira P.M.G.P. (2018). Fatigue behaviour evaluation of dissimilar polymer joints: Friction stir welded, single and double-rivets. Int. J. Fatigue.

[73] Singh R., Kumar V., Feo L., Fraternali F. (2016). Experimental investigations for mechanical and metallurgical properties of friction stir welded recycled dissimilar polymer materials with metal powder reinforcement. Compos. Part B Eng..

[74] Abdulkadhum H.H., Abdul-Khider S., Hamza S.A. (2020). Mechanical behavior of friction stir welded high-density polyethylene sheets. IOP Conf. Ser. Mater. Sci. Eng..

[75] Dashatan S.H., Azdast T., Ahmadi S.R., Bagheri A. (2013). Friction stir spot welding of dissimilar polymethyl methacrylate and acrylonitrile butadiene styrene sheets. Mater. Des..

[76] Mourad A.-H.I., Cherupurakal N., Hafeez F., Barsoum I., Genena F.A., Al Mansoori M.S., Al Marzooqi L.A. (2020). Impact strengthening of laminated kevlar/epoxy composites by nanoparticle reinforcement. Polymers.

[77] Rezaee Hajideh M., Farahani M., Molla Ramezani N. (2018). Reinforced dissimilar friction stir weld of polypropylene to acrylonitrile butadiene styrene with copper nanopowder. J. Manuf. Process..

[78] Gao J., Li C., Shilpakar U., Shen Y. (2016). Microstructure and tensile properties of dissimilar submerged friction stir welds between HDPE and ABS sheets. Int. J. Adv. Manuf. Technol..

[79] Gao J., Li C., Shilpakar U., Shen Y. (2015). Improvements of mechanical properties in dissimilar joints of HDPE and ABS via carbon nanotubes during friction stir welding process. Mater. Des..

[80] Pandey A.K., Nayak K.C., Mahapatra S.S. (2019). Characterization of friction stir spot welding between copper and poly-methyl-methacrylate (PMMA) sheet. Mater. Today Commun..

[81] Moghanian A., Paidar M., Seyedafghahi S.S., Ojo O.O. (2019). Friction stir welding of pure magnesium and polypropylene in a lap-joint configuration: Microstructure and mechanical properties. Int. J. Miner. Metall. Mater..

[82] Derazkola H.A., Kashiry Fard R., Khodabakhshi F. (2018). Effects of processing parameters on the characteristics of dissimilar friction-stir-welded joints between AA5058 aluminum alloy and PMMA polymer. Weld. World.

[83] Shahmiri H., Movahedi M., Kokabi A.H. (2017). Friction stir lap joining of aluminium alloy to polypropylene sheets. Sci. Technol. Weld. Join..

[84] Yusof F., bin Muhamad M.R., Moshwan R., bin Jamaludin M.F., Miyashita Y. (2016). Effect of surface states on joining mechanisms and mechanical properties of aluminum alloy (A5052) and Polyethylene Terephthalate (PET) by dissimilar friction spot welding. Metals.

[85] Esteves J.V., Goushegir S.M., dos Santos J.F., Canto L.B., Hage E., Amancio-Filho S.T. (2015). Friction spot joining of aluminum AA6181-T4 and carbon fiber-reinforced poly(phenylene sulfide): Effects of process parameters on the microstructure and mechanical strength. Mater. Des..

[86] Esteves J.V., Amancio-Filho S.T., Dos Santos J.F., Canto L.B., Hage E. Friction spot joining of aluminum 6181-T4 and carbon fiber reinforced poly(phenylene sulfide). Proceedings of the Annual Technical Conference of Society of Plastics Engineers.

[87] Goushegir S.M., dos Santos J.F., Amancio-Filho S.T. (2015). Influence of process parameters on mechanical performance and bonding area of AA2024/carbon-fiber-reinforced poly(phenylene sulfide) friction spot single lap joints. Mater. Des..

[88] Goushegir S.M., dos Santos J.F., Amancio-Filho S.T. (2014). Friction spot joining of aluminum AA2024/carbon-fiber reinforced poly(phenylene sulfide) composite single lap joints: Microstructure and mechanical performance. Mater. Des..

[89] Amancio-Filho S.T., Bueno C., dos Santos J.F., Huber N., Hage E. (2011). On the feasibility of friction spot joining in magnesium/fiber-reinforced polymer composite hybrid structures. Mater. Sci. Eng. A.

[90] Krishnan P.K., Christy J.V., Arunachalam R., Mourad A.-H.I., Muraliraja R., Al-Maharbi M., Murali V., Chandra M.M. (2019). Production of aluminum alloy-based metal matrix composites using scrap aluminum alloy and waste materials: Influence on microstructure and mechanical properties. J. Alloys Compd..

[91] Christy J.V., Arunachalam R., Mourad A.-H.I., Krishnan P.K., Piya S., Al-Maharbi M. (2020). Processing, Properties, and Microstructure of Recycled Aluminum Alloy Composites Produced Through an Optimized Stir and Squeeze Casting Processes. J. Manuf. Process..

[92] Arici A., Sinmaz T. (2005). Effects of double passes of the tool on friction stir welding of polyethylene. J. Mater. Sci..

[93] Arici A., Selale S. (2007). Effects of tool tilt angle on tensile strength and fracture locations of friction stir welding of polyethylene. Sci. Technol. Weld. Join..

[94] Saeedy S., Besharati Givi M.K. Experimental investigation of double side friction stir welding (FSW) on high density polyethylene blanks. Proceedings of the 10th Biennial Conference on Engineering Systems Design and Analysis.

[95] Saeedy S., Besharati Givi M.K. Experimental application of friction stir welding (FSW) on thermo plastic medium density polyethylene blanks. Proceedings of the 10th Biennial Conference on Engineering Systems Design and Analysis.

[96] Saeedy S., Besharati Givi M.K. (2011). Investigation of the effects of critical process parameters of friction stir welding of polyethylene. Proc. Inst. Mech. Eng. Part B J. Eng. Manuf..

[97] Saeedy S., Besharati Givi M.K. (2011). Experimental study on the effects of rotational speed and attack angle on high density polyethylene (HDPE) friction stir welded butt joints. Adv. Mater. Res..

[98] Abdel-Gwad E., Omar A.-B., Radwan A. (2015). Loadability of Friction Stir Welded joints of High Density Polyethylene. Int. J. Tech. Res. Appl..

[99] Husain I.M., Salim R.K., Azdast T., Hasanifard S., Shishavan S.M., Lee R.E. (2015). Mechanical properties of friction-stir-welded polyamide sheets. Int. J. Mech. Mater. Eng..

[100] Bilici M.K., Kurt B., Kurt H. (2017). Friction Stir Welded of High Density Polyethylene Sheets. J. Sci. Eng. Res..

[101] Raouache E., Boumerzoug Z., Rajakumar S., Khalfallah F. (2018). Effect of FSW process parameters on strength and peak temperature for joining high-density polyethylene (HDPE) sheets. Rev. Compos. Mater. Adv..

[102] Sheikh-Ahmad J., Ali D., Jarrar F., Deveci S. A study of friction stir welding of high density polyethylene. Proceedings of the ASME 2018 13th International Manufacturing Science and Engineering Conference.

[103] Sheikh-Ahmad J.Y., Ali D.S., Deveci S., Almaskari F., Jarrar F. (2019). Friction stir welding of high density polyethylene—Carbon black composite. J. Mater. Process. Technol..

[104] Kumar S., Medhi T., Roy B.S. (2019). Friction stir welding of thermoplastic composites. Advances in Industrial and Production Engineering, Lecture Notes in Mechanical Engineering.

[105] Rezgui M.A., Ayadi M., Cherouat A., Hamrouni K., Zghal A., Bejaoui S. Application of Taguchi approach to optimize friction stir welding parameters of polyethylene. Proceedings of the 14th International Conference on Experimental Mechanics.

[106] Rezgui M.-A., Trabelsi A.-C., Ayadi M., Hamrouni K. (2011). Optimization of Friction Stir Welding Process of High Density Polyethylene. Int. J. Prod. Qual. Eng..

[107] Moreno-Moreno M., Macea Romero Y., Rodríguez Zambrano H., Restrepo-Zapata N.C., Afonso C.R.M., Unfried-Silgado J. (2018). Mechanical and thermal properties of friction-stir welded joints of high density polyethylene using a non-rotational shoulder tool. Int. J. Adv. Manuf. Technol..

[108] Zafar A., Awang M., Khan S.R., Emamian S. (2016). Investigating friction stir welding on thick nylon 6 plates. Weld. J..

[109] Shazly M., Ahmed M.M.Z., El-Raey M. (2014). Friction stir welding of polycarbonate sheets. Minerals, Metals and Materials Society.

[110] Panneerselvam K., Lenin K. (2014). Joining of Nylon 6 plate by friction stir welding process using threaded pin profile. Mater. Des..

[111] Inaniwa S., Kurabe Y., Miyashita Y., Hori H. Application of friction stir welding for several plastic materials. Proceedings of the 1st International Joint Symposium on Joining and Welding.

[112] Aghajani Derazkola H., Simchi A. (2018). Experimental and thermomechanical analysis of friction stir welding of poly(methyl methacrylate) sheets. Sci. Technol. Weld. Join..

[113] Nandhini R., Moorthy M.K., Muthukumaran S., Kumaran S. (2019). Influence of process variables on the characteristics of friction-stir-welded polyamide 6,6 joints. Materwiss. Werksttech..

[114] Meyer S.P., Jaeger B., Wunderling C., Zaeh M.F. Friction stir welding of glass fiber-reinforced polyamide 6: Analysis of the tensile strength and fiber length distribution of friction stir welded PA6-GF30. Proceedings of the IOP Conference Series, Materials Science and Engineering.

[115] Kiss Z., Czigány T. (2007). Applicability of friction stir welding in polymeric materials. Period. Polytech. Mech. Eng..

[116] Kaddour H., Hadj Miloud M., El Bahri O., Abdellah L. (2019). Mechanical behavior analysis of a friction stir welding (FSW) for welded joint applied to polymer materials. Frat. Integrita Strutt..

[117] Sadeghian N., Besharati Givi M.K. (2015). Experimental optimization of the mechanical properties of friction stir welded Acrylonitrile Butadiene Styrene sheets. Mater. Des..

[118] Sahu S.K., Mishra D., Mahto R.P., Sharma V.M., Pal S.K., Pal K., Banerjee S., Dash P. (2018). Friction stir welding of polypropylene sheet. Eng. Sci. Technol. Int. J..

[119] Pirizadeh M., Azdast T., Rash Ahmadi S., Mamaghani Shishavan S., Bagheri A. (2014). Friction stir welding of thermoplastics using a newly designed tool. Mater. Des..

[120] Payganeh G.H., Mostafa Arab N.B., Dadgar Asl Y., Ghasemi F.A., Saeidi Boroujeni M. (2011). Effects of friction stir welding process parameters on appearance and strength of polypropylene composite welds. Int. J. Phys. Sci..

[121] Kordestani F., Ashenai Ghasemi F., Arab N.B.M. (2017). Effect of pin geometry on the mechanical strength of friction-stir-welded polypropylene composite plates. Mech. Compos. Mater..

[122] Adibeig M.R., Hassanifard S., Vakili-Tahami F., Hattel J.H. (2018). Experimental investigation of tensile strength of friction stir welded butt joints on PMMA. Mater. Today Commun..

[123] Vakili-Tahami F., Adibeig M.R., Hassanifard S. (2019). Optimizing creep lifetime of friction stir welded PMMA pipes subjected to combined loadings using rheological model. Polym. Test..

[124] Romero Y.M., Moreno Moreno M., Arrieta Cardozo B., Plata Rueda W., Consuegra Pacheco S., Unfried-Silgado J. (2018). Weldability of high-density polyethylene using friction stir welding with a non-rotational shoulder tool. Weld. Int..

[125] Zafar A., Awang M., Khan S.R., Emamian S. (2015). Effect of double shoulder tool rotational speed on thermo-physical characteristics of friction stir welded 16mm thick Nylon6. Appl. Mech. Mater..

[126] Zafar A., Awang M., Khan S.R., Emamian S. (2016). Visual analysis of material flow during friction stir welding of nylon-6. ARPN J. Eng. Appl. Sci..

[127] Mosavvar A., Azdast T., Moradian M., Hasanzadeh R. (2019). Tensile properties of friction stir welding of thermoplastic pipes based on a novel designed mechanism. Weld. World.

[128] Bagheri A., Azdast T., Doniavi A. (2013). An experimental study on mechanical properties of friction stir welded ABS sheets. Mater. Des..

[129] Moochani A., Omidvar H., Ghaffarian S.R., Goushegir S.M. (2019). Friction stir welding of thermoplastics with a new heat-assisted tool design: Mechanical properties and microstructure. Weld. World.

[130] Aydin M. (2010). Effects of welding parameters and pre-heating on the friction stir welding of UHMW-polyethylene. Polym. Plast. Technol. Eng..

[131] Banjare P.N., Sahlot P., Arora A. (2017). An assisted heating tool design for FSW of thermoplastics. J. Mater. Process. Technol..

[132] Laieghi H., Alipour S., Mostafapour A. (2020). Heat-assisted friction stir welding of polymeric nanocomposite. Sci. Technol. Weld. Join..

[133] Christy J.V., Mourad A.-H.I., Tiwai S., Sherif M.M. (2020). Influence of Graphite and Polytetrafluoroethylene Dispersions on Mechanical, Abrasive, and Erosive Wear Performance of Thermal Spray Coatings. Surf. Interfaces.

[134] Tiwari S., Christy J. (2016). V Tribological analysis of thermal spray coatings of Ni and Al2O3 with dispersion of solid lubricants in erosive wear modes. Procedia Technol..

[135] Christy J.V., Mourad A.-H.I., Tiwari S. Tribological analysis of thermal spray coatings of ni and AL2O3 with dispersion of solid lubricants in wear modes. Proceedings of the American Society of Mechanical Engineers, Pressure Vessels and Piping Division.

[136] Azarsa E., Asl A.M., Tavakolkhah V. (2012). Effect of process parameters and tool coating on mechanical properties and microstructure of heat assisted friction stir welded polyethylene sheets. Adv. Mater. Res..

[137] Mostafapour A., Azarsa E. (2012). A study on the role of processing parameters in joining polyethylene sheets via heat assisted friction stir welding: Investigating microstructure, tensile and flexural properties. Int. J. Phys. Sci..

[138] Mostafapour A., Asad F.T. (2016). Investigations on joining of Nylon 6 plates via novel method of heat assisted friction stir welding to find the optimum process parameters. Sci. Technol. Weld. Join..

[139] Mendes N., Loureiro A., Martins C., Neto P., Pires J.N. (2014). Morphology and strength of acrylonitrile butadiene styrene welds performed by robotic friction stir welding. Mater. Des..

[140] Vijendra B., Sharma A. (2015). Induction heated tool assisted friction-stir welding (i-FSW): A novel hybrid process for joining of thermoplastics. J. Manuf. Process..

[141] Nath R.K., Maji P., Barma J.D. (2019). Development of a Self-Heated Friction Stir Welding tool for welding of polypropylene sheets. J. Braz. Soc. Mech. Sci. Eng..

[142] Nateghi E., Hosseinzadeh M. (2016). Experimental investigation into effect of cooling of traversed weld nugget on quality of high-density polyethylene joints. Int. J. Adv. Manuf. Technol..

[143] Arunachalam R., Piya S., Krishnan P.K., Muraliraja R., Christy J.V., Mourad A.-H.I., Al-Maharbi M. (2020). Optimization of stir–squeeze casting parameters for production of metal matrix composites using a hybrid analytical hierarchy process–Taguchi-Grey approach. Eng. Optim..

[144] Muñoz S.A. Friction stir welding FSW of gas polyethylene pipes. Proceedings of the 6th Pan American Conference for NDT.

[145] Christy J.V., Mourad A.-H.I., Arunachalam R. Mechanical and Tribological Evaluation of Aluminum Metal Matrix Composite Pipes Fabricated by Gravity and Squeeze Stir Casting. Proceedings of the ASME 2019 Pressure Vessels & Piping Conference; American Society of Mechanical Engineers Digital Collection.

[146] Christy J.V., Mourad A.-H.I., Arunachalam R. Sustainable Manufacturing and Optimization of Squeeze Stir Cast Rods Using Recycled Aluminum and Alumina Reinforcements. Proceedings of the Pressure Vessels & Piping Conference.

[147] Ahmadi H., Arab N.B.M., Ghasemi F.A., Farsani R.E. (2012). Influence of pin profile on quality of friction stir lap welds in carbon fiber reinforced polypropylene composite. Int. J. Mech. Appl..

[148] Ahmadi H., Mostafa Arab N.B., Ghasemi F.A. (2014). Optimization of process parameters for friction stir lap welding of carbon fibre reinforced thermoplastic composites by Taguchi method. J. Mech. Sci. Technol..

[149] Aghajani Derazkola H., Simchi A. (2018). Experimental and thermomechanical analysis of the effect of tool pin profile on the friction stir welding of poly(methyl methacrylate) sheets. J. Manuf. Process..

[150] Gao J., Shen Y., Zhang J., Xu H. (2014). Submerged friction stir weld of polyethylene sheets. J. Appl. Polym. Sci..

[151] Yan Y., Shen Y., Lan B., Gao J. (2017). Influences of friction stir welding parameters on morphology and tensile strength of high density polyethylene lap joints produced by double-pin tool. J. Manuf. Process..

[152] Eslami S., Ramos T., Tavares P.J., Moreira P.M.G.P. (2015). Effect of friction stir welding parameters with newly developed tool for lap joint of dissimilar polymers. Procedia Eng..

[153] Eslami S., Ramos T., Tavares P.J., Moreira P.M.G.P. (2015). Shoulder design developments for FSW lap joints of dissimilar polymers. J. Manuf. Process..

[154] Elyasi M., Derazkola H.A. (2018). Experimental and thermomechanical study on FSW of PMMA polymer T-joint. Int. J. Adv. Manuf. Technol..

[155] Lambiase F., Paoletti A., Di Ilio A. (2015). Mechanical behaviour of friction stir spot welds of polycarbonate sheets. Int. J. Adv. Manuf. Technol..

[156] Lambiase F., Paoletti A., Di Ilio A. (2017). Friction spot stir welding of polymers: Control of plunging force. Int. J. Adv. Manuf. Technol..

[157] Bilici M.K., Yükler A.I., Kurtulmuş M. (2011). The optimization of welding parameters for friction stir spot welding of high density polyethylene sheets. Mater. Des..

[158] Bilici M.K. (2012). Application of Taguchi approach to optimize friction stir spot welding parameters of polypropylene. Mater. Des..

[159] (2012). Memduh Kurtulmus Friction stir spot welding parameters for polypropylene sheets. Sci. Res. Essays.

[160] Tasdemir M., Bilici M.K., Kurt M. (2016). Relation between friction stir spot welding parameters and mechanical properties of high density polyethylene/glass spheres polymer composites. Mater. Sci. Forum.

[161] Yan Y., Shen Y., Zhang W., Hou W. (2018). Friction stir spot welding ABS using triflute-pin tool: Effect of process parameters on joint morphology, dimension and mechanical property. J. Manuf. Process..

[162] Lambiase F., Paoletti A., Di Ilio A. (2017). Effect of tool geometry on mechanical behavior of friction stir spot welds of polycarbonate sheets. Int. J. Adv. Manuf. Technol..

[163] Bilici M.K. (2012). Effect of tool geometry on friction stir spot welding of polypropylene sheets. Express Polym. Lett..

[164] Bilici M.K., Yükler A.I. (2012). Influence of tool geometry and process parameters on macrostructure and static strength in friction stir spot welded polyethylene sheets. Mater. Des..

[165] Bilici M.K., Yukler A.I., Kurtulmus M., Kastan A. (2018). The Optimization of Welding Tool Material and Welding Parameters in Friction Stir Spot Welding of Plastics Using Taguchi Experimental Design. Int. J. Eng. Sci. Appl..

[166] Mozumder M.S., Mairpady A., Mourad A.-H.I. (2019). Hdpe/Tio2 nanocomposite: Fabrication and optimization of mechanical property by RSM and ANN. Solid State Phenomena.

[167] Thekkuden D.T., Mourad A.-H.I., Christy J.V., Idrisi A.H. Assessment of Weld Quality Using Control Chart and Frequency Domain Analysis. Proceedings of the Pressure Vessels & Piping Conference.

[168] Eslami S., Miranda J.F., Mourão L., Tavares P.J., Moreira P.M.G.P. (2018). Polyethylene friction stir welding parameter optimization and temperature characterization. Int. J. Adv. Manuf. Technol..

[169] Stan F., Stanciu N.V., Fetecau C., Sandu L.I. Characterization of welding attributes in friction spot stir welding of high-density polyethylene/multi-walled carbon nanotube composites. Proceedings of the 13th International Manufacturing Science and Engineering Conference.

[170] Kurtulmuş M., Kiraz A. (2018). Artificial neural network modelling for polyethylene FSSW parameters. Sci. Iran..

[171] Chavan A., Shete M.T. (2015). Optimization of Friction Stir Spot Welding Process using Artificial Neural Network. Int. J. Sci. Technol. Eng..

[172] Paoletti A., Lambiase F., Di Ilio A. (2015). Optimization of friction stir welding of thermoplastics. Procedia CIRP.

[173] Babaghayou M.I., Mourad A.-H.I., Lorenzo V., Chabira S.F., Sebaa M. (2018). Anisotropy evolution of low density polyethylene greenhouse covering films during their service life. Polym. Test..

[174] Babaghayou M.I., Mourad A.-H.I., Lorenzo V., de la Orden M.U., Martínez Urreaga J., Chabira S.F., Sebaa M. (2016). Photodegradation characterization and heterogeneity evaluation of the exposed and unexposed faces of stabilized and unstabilized LDPE films. Mater. Des..

